# Borneol-driven meningeal lymphatic drainage clears amyloid-β peptide to attenuate Alzheimer-like phenotype in mice

**DOI:** 10.7150/thno.76133

**Published:** 2023-01-01

**Authors:** Yue Wu, Tingting Zhang, Xianqiang Li, Yimei Wei, Xinyu Li, Sixue Wang, Jun Liu, Dan Li, Shujun Wang, Tiantian Ye

**Affiliations:** 1 Department of Pharmaceutics, College of Pharmacy, Shenyang Pharmaceutical University, Shenyang, 110016, China.; 2 Department of Pharmaceutics, Wuya College of Innovation, Shenyang Pharmaceutical University, Shenyang Junhong Pharmaceutical Technology Co., Ltd. Shenyang, 110016, China.; 3 Research and development center, Jiangsu Aidi Nano Biomedical Co., Ltd., Nantong, 226000, China.; 4 Department of Pharmaceutics, College of Chinese Pharmacy, Shenyang Pharmaceutical University, Shenyang, 110016, China.; 5 Research and development center, Shenyang Junhong Pharmaceutical Technology Co., Ltd. Shenyang, 110031, China.

**Keywords:** Borneol, meningeal lymphatic vessels, lymphatic clearance, amyloid-β peptide, Alzheimer's disease

## Abstract

**Rationale:** The accumulation and clearance of amyloid-β (Aβ) peptides play a crucial role in the pathogenesis of Alzheimer's disease (AD). The (re)discovery of meningeal lymphatic vessels in recent years has focused attention on the lymphatic clearance of Aβ and has become a promising therapeutic target for such diseases. However, there is a lack of small molecular compounds that could clearly regulate meningeal lymphatic drainage to remove Aβ from the brain.

**Methods:** We investigated the effect of borneol on meningeal lymphatic clearance of macromolecules with different molecular weights (including Aβ) in the brain. To further investigate the mechanism of borneol regulating meningeal lymphatic drainage, immunofluorescence staining, western blotting, ELISA, RT-qPCR, and Nitric Oxide assay kits were used. The cognitive function of AD mice after borneol treatment was evaluated using two behavioral tests: open field (OF) and Morris water maze (MWM).

**Results:** This study discovered that borneol could accelerate the lymphatic clearance of Aβ from the brain by enhancing meningeal lymphatic drainage. Preliminary mechanism analysis revealed that borneol could improve the permeability and inner diameter of lymphatic vessels, allowing macromolecules to drain into the cervical lymph nodes (CLNS) and then be transported to the lymphatic circulation. To speed up the clearance of macromolecules, borneol also stimulated lymphatic constriction by lowering the level of nitric oxide in the meninges. In addition, borneol stimulated lymphangiogenesis by increasing the levels of FOXC2, VEGFC, and LYVE-1 in the meninges, which promoted the clearance rates of macromolecules. Borneol improved meningeal lymphatic clearance not only for Aβ but also for other macromolecular polymers (molecular weight in the range of 2 KD - 45 KD. Borneol ameliorated cognitive deficits and alleviated brain Aβ burden in Aβ-injected mice.

**Conclusions:** Our findings not only provide a strategy to regulate lymphatic clearance pathways of macromolecules in the brain, but also new targets and ideas for treating neurodegenerative diseases like AD. Furthermore, our findings indicate that borneol is a promising therapeutic drug for AD.

## Introduction

Alzheimer's disease (AD) is the most common form of neurodegenerative disorder accounting for 60% to 80% of all dementia cases and affecting close to 15 million people worldwide as its prevalence increases with age 1,2. The accumulation and aggregation of amyloid-beta (Aβ) peptides are one of the neuropathological trademarks of AD. According to mounting evidence, an imbalance in Aβ production and clearance is a major cause of Aβ aggregation and accumulation in the brain. Progressive impairment of Aβ clearance mechanisms is a common prelude to late-onset AD, which results in cognitive impairment and behavioral deficits 3-5. Because there are no effective treatments for AD, current studies are focused on preventing the accumulation and migration of Aβ aggregates in the blood and lymph vasculature 6. Previous research has demonstrated a close association between Aβ clearance and the meningeal lymphatic system. Aβ accumulation may induce lymphatic dysfunction, resulting in a positive feedback loop that decreases Aβ clearance 7-9. Nevertheless, the highly anticipated Aβ clearance immunotherapy was defeated in recent clinical trials because of the complexity of the AD mechanism 10, indicating the urgent need for a novel Aβ clearance strategy.

The (re)discovery of meningeal lymphatic vessels has encouraged a re-assessment of processes in developing numerous neurodegenerative disorders, the most common being AD 7,11-13. To maintain brain homeostasis 14, lymphatic vessels in the meninges drain cerebrospinal fluid (CSF) macromolecules into the cervical lymph nodes (CLNs). These lymphatics, as essential physiological regulators, play a role in fluid drainage, immune surveillance, and disease progression in AD 8,15. To alleviate cognitive impairment and behavioral deficits in transgenic mouse models of AD, the meningeal lymphatic channels could be assisted to drain extracellular Aβ from the brain into deep cervical lymph nodes (dCLNs) 16. When the meningeal lymphatic system is dysfunctional by ablation of meningeal lymphatic vessels 8 or ligation of cervical lymph vessels, the accumulation of Aβ in the brain is accelerated 17. Enhancement of meningeal lymphatic drainage by vascular endothelial growth factor C (VEGFC), DSCR1, immunotherapy, and physical stimulation treatment to accelerate Aβ clearance in the brain could ameliorate cognitive dysfunction in an aging mouse model of amyloid pathology (5XFAD) 16,18-20. However, these lymphatic drainage improvement strategies may be difficult to achieve clinically due to high off-targets and low controllability 7,16. Therefore, more drug strategies for effectively regulating meningeal lymphatic drainage and enhancing the transport capacity of meningeal lymphatic vessels to remove Aβ in the brain are required.

Borneol (C_10_H_18_O, molecular weight, 154.25), a highly lipid-soluble bicyclic naturally bicyclic monoterpene component, could quickly cross the BBB and enter the brain after oral administration for 5 min, and reach the maximum concentration in the brain within 1 h 21,22, which is distributed highest in the cortex, moderate in the hippocampus and hypothalamus, and the lowest in the striatum 23,24. Increasing evidence suggests that borneol has multiple bioactivities in stroke, AD, and epilepsy, such as neuroprotection, anti-inflammatory, and anti-epileptogenic 25-27. The mechanism of borneol in the treatment of brain illness involves multiple effects, including reducing acetylcholinesterase (AChE) and enhancing cholinergic transmission in the brain of APP/PS1 transgenic mice 28 and up-regulating vascular endothelial growth factor to improve local microcirculation 29. Nonetheless, no reports of borneol regulating meningeal lymphatic drainage have been found. We discovered that borneol can improve the lymphatic-targeting ability of large molecules and increase lymph node uptake for the first time 30. As a result, we hypothesized that after oral administration, borneol would rapidly enter the brain and promote the drainage of macromolecules such as Aβ into the meningeal lymphatic system. It would be a significant breakthrough that borneol could accelerate Aβ lymphatic clearance by improving meningeal lymphatic drainage.

In this study, we looked at how borneol affected meningeal lymphatic drainage to clear Aβ in the central nervous system (CNS). Firstly, we found that oral administration of borneol micelles (BO-Ms) could enhance the transport of macromolecules injected into the CNS by meningeal lymphatic vessels. When afferent lymphatic vessels to dCLNs were ligated, the efflux of macromolecules into dCLNs was significantly reduced by BO-Ms. BO-Ms facilitated the outflow of Aβ from the brain via meningeal lymphatic vessels, which was dependent on the improvement of meningeal lymphatic function and the expression of lymphangiogenesis proteins. Our results showed that BO-Ms increase Aβ lymphatic clearance via meningeal lymphatic vessels and alleviate amyloid pathology, implying a promising drug for AD treatment.

## Materials and Methods

### Materials

The borneol was provided by Hubei YuanCheng Saichuang Technology Co., Ltd. (Hubei, China). Ovalbumin-FITC (OVA-FITC) was obtained from Beijing Bersee® Technology Co., Ltd. Aβ_42_ and Aβ_42_-FITC were purchased from Nanjing Peptide Biotech Ltd. CY5.5 was purchased from Xi'an Kaixin Biotechnology Co., Ltd. MPEG2000-PDLLA2000 was obtained by Jinan Daigang Biomaterial Co., Ltd. Indocyanine green (ICG) was purchased from Dalian Meilun Biotechnology Co., Ltd. Aβ_42_ ELISA Kit (JL11012) and VEGFC ELISA kit (JL12038) were achieved from the Shanghai Jianglai industrial Limited by Share Ltd. Mouse FOXC2 ELISA Kit was purchased from Animalunion Biotechnology Co., Ltd. Nitric Oxide (NO) assay kit (A012-1) was obtained from Nanjing Jiancheng Bioengineering Institute, China. The following antibodies were applied for OCT-embedded sections: Anti-LYVE-1 (rabbit, 1:250, Affinity, AF4202), Anti- Podoplanin (rabbit, 1:200, Affinity, bs-1048R), Anti-Aβ1-42 antibody (rabbit, 1:200, Affinity, bs-23379R), Goat Anti-Rabbit IgG (H+L) CY3-conjugated (1:1000, Affinity, S0011), Alexa Fluor® 488-conjugated Goat Anti-Rabbit IgG (H+L) (1:400, Servicebio, GB25303), IgG (H+L) (Cy5 conjugated Goat Anti-mouse IgG (H+L)) (1:400, Servicebio, GB27301), Alexa Fluor 488-conjugated anti-LYVE-1 antibody (Rabbit anti-LYVE-1/AF488) (Cat. No. bs-1311R-AF488, Bioss). The antibodies for western blot analysis were included Anti-VEGF-C antibody (rabbit, 1:500, Affinity, bs-1586R), Anti-FOXC-2 antibody (rabbit, 1:500, Affinity, bs-8730R), Rabbit Anti-beta-Actin (Loading Control), Polyclonal Antibody (1:1000, Bioss, cat no. bs-0061R), and Rabbit Anti-HPR antibody (1:2000, Bioss, cat no. bs-9912R). MLECs were purchased from Qingqi (Shanghai) Biotechnology Development Co., Ltd. All other chemicals and reagents were of analytical grade and used without further purification.

### Animals

Male Kunming mice (6-8 weeks old) and Sprague-Dawley (SD) rats were purchased from the Laboratory Animal Center, Shenyang Pharmaceutical University (Shenyang, China). Mice and rats were housed under standard environmental conditions with controlled temperature and habituation, on a 12 h/12 h light/dark cycle, and fed with regular water and food. All the animal experimental protocols and procedures were approved by the Experimental Animal Use and Care Committee, Shenyang Pharmaceutical University, and followed the Guiding Principles for the Care and Use of Laboratory Animals approved by the Animal Regulations of the National Science and Technology Committee of China.

### Formulation preparation

#### Preparation and characterization of blank micelles (BL-Ms)

For the preparation of BL-Ms, propylene glycol, PEG-400, and tween 80 were mixed and then dropped into the distilled water under stirring. Then the particle size and zeta potential of BO-Ms were investigated using a Zetasizer Nano-ZS90 (Malvern Instruments Ltd., Malvern, UK) at 25 °C.

#### Preparation and characterization of BO-Ms

The borneol was ground and sieved by the 200-mesh sieve. The borneol powders were dissolved in propylene glycol and thoroughly mixed with PEG-400 and tween 80 before being diluted with the distilled water. The borneol suspensions were sonicated until clear. The morphologies of BO-Ms were observed using transmission electron microscopy (JEM-1200EX, JEOL, Inc., Tokyo, Japan). The particle size and Zeta potential of BO-Ms were assayed by laser light scattering using a Zetasizer Nano-ZS90 (Malvern Instruments Ltd., Malvern, UK) at 25 °C.

#### Preparation of CY5.5 Micelles (CY5.5-Ms)

Briefly, a mixture of CY5.5/MPEG2000-PDLLA2000 at a ratio of 1:20 was dissolved in chloroform and dried into a thin film by a rotary evaporator (RE52CS; Shanghai Yarong Bio-Chem Instruments, Shanghai, China). The film was hydrated with saline in a water bath at 60 °C. The CY5.5-Ms were filtered using a 0.8 μm filter membrane. The absorbance (OD) of CY5.5 was measured at the excitation wavelength of 610 nm and emission wavelength of 700 nm in a microplate reader (SpectraMax M3, Molecular Devices, USA). The fluorescence emission spectra of CY5.5-Ms was detected by a Thermo Scientific Varioskan Flash. The particle size of CY5.5-Ms was assayed by laser light scattering using a Zetasizer Nano-ZS90 (Malvern Instruments Ltd., Malvern, UK) at 25 °C.

#### Preparation of ICG nanoparticles (ICG-NPs)

ICG was dissolved in methanol and slowly added to the aqueous solution of hyaluronic acid (HA) - octadecylamine (OA), and then dialyzed in purified water for 12 h to remove the solvent and unpackaged drugs. Finally, the solution was frozen dry. The fluorescence of ICG-NPs was visualized by a near-infrared fluorescence imaging system of DPM. The fluorescence emission spectra of ICG-NPs were detected by a Thermo Scientific Varioskan Flash. The particle size of ICG-NPs was assayed by laser light scattering using a Zetasizer Nano-ZS90 (Malvern Instruments Ltd., Malvern, UK) at 25 °C.

#### Preparation of Aβ_42_ oligomers / Aβ_42_-FITC oligomers / Aβ_42_ fibrillation

The preparation for Aβ_42_ oligomers and fibrillation was performed as described before 31,32. In short, 1 mg Aβ_42_/Aβ_42_-FITC peptide was dissolved in 0.2 mL of HFIP and incubated at room temperature for 24 h. Then, HFIP was removed by evaporation under a gentle stream of nitrogen gas.

For Aβ_42_ / Aβ_42_-FITC oligomers, Aβ_42_/Aβ_42_-FITC peptides were redissolved with 100 μL of DMSO and diluted with 2.1 mL of pure water. The Aβ peptide solution was incubated at 4 °C for 24 h and then centrifuged at 4 °C and 14000 r/min for 10 min. The supernatant was taken as Aβ_42_ oligomers/Aβ_42_-FITC oligomers.

For Aβ_42_ fibrillation, Aβ_42_ peptide was prepared by dissolving in DMSO and then diluting with aqueous 10 mmol /L HCl containing 140 mm NaCl and 2.7 mm KCl. Then the Aβ_42_ peptide solution was incubated at 37 °C for 7 d.

The final concentration of Aβ_42_ oligomers and fibrillation was ~50 μM. Aβ_42_ oligomers and fibrillation were stored in a refrigerator at -80 °C.

### An animal model with lateral ventricle injection (i.c.v.)

Mice were anesthetized by i.p. injection of 1% pentobarbital sodium, and the head was fixed in a stereotactic frame. An incision was made in the skin after shaving the head to expose the skull. A hole was drilled at -0.3 mm in the anterior-posterior axis and +1.0 mm in the medial-lateral axis relative to the bregma. 5 μL of either FITC conjugated OVA (at 2 mg/mL), CY5.5-Ms, FITC conjugated Aβ_42_ (~50 μmol) oligomers, Aβ_42_ oligomers (~50 μmol), or ICG-NPs (5 mg/mL) were injected at a rate of 1 μL/min into the brain parenchyma by using a Hamilton syringe. After injecting, the syringe was in place for a further 5 min to prevent backflow. Then the scaled skin was sutured, and the mice were subcutaneously injected with ketoprofen (2 mg/Kg). Afterward, mice in the BO-Ms group were orally administrated with 20 mg/kg BO-Ms. The mice regained consciousness on a 37 °C pad until further use.

### An animal model with hippocampus injection

Mice were anesthetized by i.p. injection of 1% pentobarbital sodium, and the head was fixed in a stereotactic frame. After shaving the head, an incision was made in the skin to expose the skull. Five microliters of OVA-FITC or ICG-NPs were stereotactically injected at -2.5 mm in the anterior-posterior axis and +2.2 mm in the medial-lateral axis relative to the bregma, respectively. After injecting, the syringe was in place for a further 5 min to prevent backflow. Then the scaled skin was sutured, and the mice were subcutaneously injected with ketoprofen (2 mg/Kg). The mice were taken orally with 20 mg/kg BO-Ms and regained consciousness on a 37 °C pad until further use.

### Intra-cisterna magna injections (i.c.m injection)

Mice were anesthetized by intraperitoneal (i.p.) injection of sodium pentobarbital (50 mg/kg). The iodine and 70% ethanol were used to clean the skin of the neck. Mice were secured in a stereotaxic frame (RWD), and an incision was done along with separation of the posterior neck skin and muscles to expose the cisterna magna. Then 3 μL of Alexa Fluor 488-conjugated anti-LYVE-1 antibody (Anti-LYVE-1/AF488) (Cat. No. bs-1311R-AF488, Bioss) was injected into the cisterna magna at a rate of 1 µL/min. 5 µL even blue (EB) or ICG-NPs at the concentration of 5 mg/mL were injected into the cisterna magna at a speed of 1 μL/min, respectively. After injection, the needle was left in place for 2 min to prevent backflow and leakage of CSF. The neck skin was sutured, and the mice were injected with ketoprofen (2 mg/Kg) by subcutaneous injection. Afterward, mice were given BO-Ms (20 mg/kg) via oral gavage and allowed to recover on a 37 °C heating pad until responsive.

### Lymphatic vessel ligation

Mice were anesthetized with 1% pentobarbital sodium (i.p.), shaved, and cleaned at the neck with iodine and 70% ethanol. A midline incision was made 5 mm superior to the clavicle. The muscles and fascia were carefully separated, and the dCLNs were exposed on each side. For ligation, the afferent lymphatic vessels anterior to the dCLNs on each side were ligated using 10-0 nylon nonabsorbable sutures. Sham-operated mice were only submitted to skin incision and muscles and fascia separation to expose dCLNs without ligation. Mice were then sutured and injected with analgesics and antibiotics subcutaneously. Mice were allowed to recover on a heating pad until conscious.

### Tissue collection and processing

For tissue analysis, mice were given a lethal dose of pentobarbital sodium. The brain, dCLNs, and blood were immediately collected and stored at -20 °C until analysis. After puncturing the right auricle, mice perfused through the left ventricle with ice-cold PBS, followed by 4% paraformaldehyde (PFA) for 10 min.

The whole brain and dCLNs were dissected and then dropped fixed in 4% PFA at 4 °C, incubated in 30% sucrose solutions, and frozen in Tissue-Plus® O.C.T. compound (Thermo Fisher Scientific). Then, brains or dCLNs were sliced (20 or 10 μm thick sections) with a cryostat (Thermo Fisher), and sections were immediately collected into Superfrost™ Plus slides and kept at -80 °C until further use. After transcardial perfusion with saline and 4% PFA for 10 min, the skullcap was harvested and preserved in 4% PFA at 4 °C. Meninges were carefully dissected from the skullcaps with Dumont #5 forceps. For cribriform plate tissues, mice were then decapitated, and the skin of the entire head was removed using forceps and scissors to separate the skin from the muscles and ear canal. The whole heads were fixed in 4% PFA for 24 h, then the heads were decalcified for 60 h in formic acid (4%) solution and saturated in 30% sucrose. The formic acid (4%) solution was replaced each 12 h. Tissue frozen sections of 50 μm thich were obtained on a Slee MNT cryostat, mounted on Premiere microscope slides, and stored at -20 °C. For lymphatic vessels in the leg of rats, 100 μL BO-Ms with the concentration of 2 mg/mL were subcutaneously injected into the right rear footpad of SD rats. After 30 min, the lymphatic vessels were removed and dropped fixed in 4% PFA. Then, 10 μm thick sections were sliced with a cryostat (Thermo Cryotome E), sections were immediately collected into Superfrost™ Plus slides and kept at -80 °C until further use.

### Aβ_42_ measurement in tissues and blood

Brain and dCLNs for ELISA were rinsed with ice-cold 10 mM pH 7.4 PBS to remove residual blood and minced after weighing. The chopped tissue was mixed with 10 mM pH 7.4 PBS containing protease inhibitor at a ratio of 1:9 (weight/volume, g/mL) in a glass homogenizer. Then the tissue was fully ground on ice and further disrupted by ultrasonication. The homogenate was centrifuged at 5000 g for 10 min, and the supernatant was removed for ELISA measurement of soluble Aβ_42_ without further dilution.

### ELISA for VEGFC and FOXC2

VEGFC and FOXC2 content in meninges of rats were measured in duplicate determinations with a commercially available ELISA.

### Quantitative RT-PCR

The mRNA levels of VEGFC and FOXC2 in the meninges of rats were performed by the RT-qPCR assay. Briefly, the rats were given 14 mg/kg BO-Ms orally, and the rats in the control group were given the same amount of normal saline. After 30 min, the meninges of the rats in each group were removed. Total RNA was extracted using RNA extraction according to the manufacturer's instructions. A reverse transcription kit was used to synthesize the complementary DNA. Then real-time quantitative PCR (qPCR) was performed and relative mRNA expression levels were studied through the 2ΔΔCt method. The primers used were listed in Table S1.

### Immunohistochemistry

For immunofluorescence, mouse brain sections, dCLNs sections, meningeal wholemounts, cribriform plate sections, and lymphatic vessels were rinsed in 10 mM pH 7.4 PBS, blocked with 1% Triton-X-100 in PBST for 10 min, and blocked in 2% bovine serum albumin (BSA) for 1 h at RT, then incubated with primary antibodies diluted in PBS with 2% BSA and 1% Triton X-100 at 4 °C overnight. The sections were washed with PBS three times for 15 min each and incubated with secondary antibodies (diluted in PBS with 2% BSA and 1% Triton X-100) at room temperature for 2 h. Finally, the sections and whole mounts were washed and mounted mounting medium with 4,6-diamidino-2-phenylindole. Images were acquired either using a widefield microscope (Leica) or a confocal microscope (Nikon). The primary antibodies were used in immunofluorescence included Anti-LYVE-1 (rabbit, 1:250, Affinity, AF4202) and Anti-Podoplanin (rabbit, 1:200, Affinity, bs-1048R), Anti-Aβ1-42 antibody (rabbit, 1:200, Affinity, bs-23379R). The corresponding secondary antibodies were used as follows: Goat Anti-Rabbit IgG (H+L) CY3-conjugated (1:1000, Affinity, S0011), Alexa Fluor® 488-conjugated Goat Anti-Rabbit IgG (H+L) (1:400, Servicebio, GB25303), IgG (H+L) (Cy5 conjugated Goat Anti-mouse IgG (H+L)) (1:400, Servicebio, GB27301).

### Imaging and quantifications

The images of whole-mount staining meninges were acquired under the 10× lens of a Leica DM4 B with a resolution of 1024×1024 resolution and a z-step of 4 µm. Image J software was used to perform quantitative evaluations of the micrographs. Images of the same region of the superior sagittal sinus (SSS), the confluence of sinuses (COS), and the transverse sinus (TS) were acquired in a confocal microscope. The means of 30 individual lymphatic vessel diameter measurements. The percentage of meningeal lymphatics labeled by AF488 LYVE-1 antibody (i.c.m.) was defined by dividing the area of AF488 LYVE-1 antibody (i.c.m.) labeled by the area of meningeal lymphatics. Fluorescent stereomicrographs of labeled skullcap were obtained with Leica. To quantify the number of total OVA-FITC or Aβ_42_-FITC in the brain, the images of brain sections were obtained using the confocal microscope. The area of OVA-FITC or Aβ_42_-FITC was determined by dividing the area labeled OVA or Aβ_42_ per section by the area of the brain section. The area of OVA-FITC or Aβ_42_-FITC in the dCLNs was also calculated with the same method. The area of LYVE-1 near the cribriform plate was determined by dividing the area of labeled LYVE-1 per section by the area of the cribriform plate section. All fluorescence micrographs were managed with equally constant exposure time and brightness/contrast and analyzed using the Image J software.

### Western-Blot assay

Western blot was applied to analyze the lymphoid-associated protein changes in meninges at different time points after BO-Ms (20 mg/kg) were treated. The meninges were harvested after mice euthanasia and perfusion with cold PBS. The total protein was extracted with RIPA lysis buffer and 1% PMSF. Then, the PVDF membranes were blocked and incubated with one of the following primary antibodies: Anti-LYVE-1 (rabbit, 1:1000, Affinity, AF4202), Anti-VEGF-C antibody (rabbit, 1:500, Affinity, bs-1586R), Anti-FOXC-2 antibody (rabbit, 1:500, Affinity, bs-8730R), and Rabbit Anti-beta-Actin (Loading Control) Polyclonal Antibody (1:1000, Bioss, cat no. bs-0061R) overnight at 4 °C. The membranes were sufficiently washed and incubated with horseradish peroxidase-conjugated secondary antibodies (1:2000, Bioss, cat no. bs-9912R) for 2 h at room temperature. The membranes were visualized using an Amersham Imager 680, and the grey values of the bands were calculated by Image J software.

### *In vivo* imaging study

Following the intravenous delivery of OVA-FITC into the brain, saline or BO-Ms at doses of 20 mg/kg were administered right away. At 10 min, 30 min, 60 min, and 120 min oral administration, the mice were euthanasia, and brain and dCLNs were removed. The pseudo-color fluorescence images of the brain were detected by *in vivo* imaging system (Carestream Image Station System FX Pro, Carestream Health, Inc., NY, USA), in which the excitation wavelength and emission wavelength were 470 nm and 535 nm, respectively.

### Indocyanine green near-infrared (ICG-NIR) imaging

ICG-NPs lyophilized powder was dissolved in saline (5 mg/mL). The mice from the BO-Ms group and the saline group were anesthetized and fixed in a stereotactic frame (RWD). 5 μL ICG-NPs were stereotactically injected at the position at lateral ventricles or hippocampus (1 μL /min), and then the needle was left in place for 3 min to avoid leakage. Mice in the BO-Ms group were orally administrated with 20 mg/kg BO-Ms, and mice in the control group were orally administrated with the same amount of normal saline. ICG fluorescence of dCLNs and their afferent lymphatics were visualized and recorded by the near-infrared fluorescence imaging system of DPM for 4 h.

### NO analysis

The lymph flow function in the meninges was estimated by NO levels. Briefly, mice were oral administration of BO-Ms at dosages of 20 mg/kg, and the mice in the control group were orally given the same amount of normal saline. At the time point of 10 min, 30 min, and 60 min, the meninges were harvested. The meninges in PBS buffer containing protease inhibitors were homogenized and centrifuged to obtain the supernatant. The expression levels of NO in tissues were measured by using the Nitric Oxide assay kits (Nanjing Jiancheng Biotechnology Institute, China), and the specific procedures were strictly by the instructions of the kit.

### Proliferation assay of lymphatic endothelial cells

MLECs were seeded in 96-well plates at a density of 5×10^3^ cells/well. After allowing them to grow for 12 h, a medium containing different concentrations (0, 1, 2, 5, 10, 20, 50 μg/mL) of borneol was added. After another 24 h of culture, we added 10 μL of CCK-8 reagent. Plates were incubated at 37 °C for 1.5 h and measured by recording the absorbance at 450 nm with a microplate reader (Multiskan MK3, Thermo, MA, USA).

### Transwell assay of lymphatic endothelial cells

MLECs (2×10^5^ cells/mL) in 100 μL of FBS-free DMEM were seeded into the upper chambers of a transwell plate. Then 500 μL borneol at the concentration of 0, 20, and 50 μg/mL were added to the bottom chambers. The plate was incubated at 37 °C for 6 h, and cells were fixed in 4% PFA and labeled with 0.1% crystal violet. Then the stained migrating cells were observed microscopically.

### Surgical procedures and experimental groups for therapy of AD mice

Male KM AD mice (4 months) were established through hippocampus injection of Aβ_42_ according to the literature 33,34, but with modifications. Mice were anesthetized by i.p. injection of 1% pentobarbital sodium, and the head was fixed in a stereotactic frame. An incision was made in the skin after shaving the head to expose the skull. Three microliters of the aggregated form of Aβ_42_ were injected into the CA1 regions on both sides of the hippocampus (from bregma: AP: - 2.3 mm, ML: ± 1.8 mm, DV: -2.0 mm), respectively. After injecting, the syringe was in place for a further 5 min to prevent backflow. Then the scaled skin was sutured, and the mice were subcutaneously injected with ketoprofen (2 mg/Kg).

Mice injected with Aβ_42_ were randomly subdivided into two groups with six animals per group: the AD group and the BO-Ms group. Mice in the BO-Ms group were given BO-Ms (20 mg/kg) via oral gavage every day for 14 consecutive days. AD mice and control mice were orally administered saline in a volume of 0.01 mL/g of mouse weight daily for 14 d. Mice in each group were tested by a series of behavioral studies after drug treatment.

### Open field test (OF)

Mice were carried to the behavior room at least 30 min before starting the test to habituate to the environment. Mice were then placed into the open field arena (made of opaque white plastic material, 25 cm × 25 cm) by a blinded experimenter and allowed to explore it for 15 min. Total distance (in mm) and % time spent in the center (8 cm × 8 cm) were quantified using video software (ZH-ZFT, Anhui Zhenghua Biologic Apparatus Facilities Co., Ltd, China).

### Morris water maze test (MWM)

Mice were carried to the behavior room at least 30 min prior to starting the test to habituate to the environment. The MWM test consisted of 4 d of acquisition and 1 d of probe trial. The maze was filled with water, and the water temperature was kept at 22 ± 1 °C. The maze contained fixed visual cues on each quadrant's walls. For the acquisition trial, mice performed four trials every day for 4 consecutive days to find a hidden platform (10 cm diameter) located 1 cm below the water surface in a pool 1.2 m in diameter. The latency is the mean time required by the mouse to find and climb onto the platform starting from different quadrants, recorded for up to 60 s. Each mouse was given a 15 s respite on the platform before being transferred from the maze to its own cage. If the mouse failed to find the platform within 60 s, it was guided to remain on the platform for 15 s. After removing the platform from the water on the fifth day, each mouse's spatial memory was examined in a probe trial for 60 s. Data were recorded using the ZH-Morris image motion system (Anhui Zhenghua Biologic Apparatus Facilities Co., Ltd, China) in the acquisition and probe trial. The time that each mouse searched for the platform in the target quadrant, the number of times crossing the platform, and the speed were recorded.

### Statistical analysis

Mice were randomly assigned to the group. The number of animals shown in each figure is represented in the legend as n = x mice per group, and the data is presented as multiple measurements per animal. All data was performed as mean ± standard error mean. Statistical analysis was performed using Prism software (GraphPad). The statistical significance between three or more groups with one experimental parameter was assessed by One-way ANOVA, while between groups with two experimental conditions was evaluated by two-way ANOVA. More information on statistical validation saw legends.

## Results

### BO-Ms facilitate the clearance of macromolecules in the CNS

**Figure 1:**
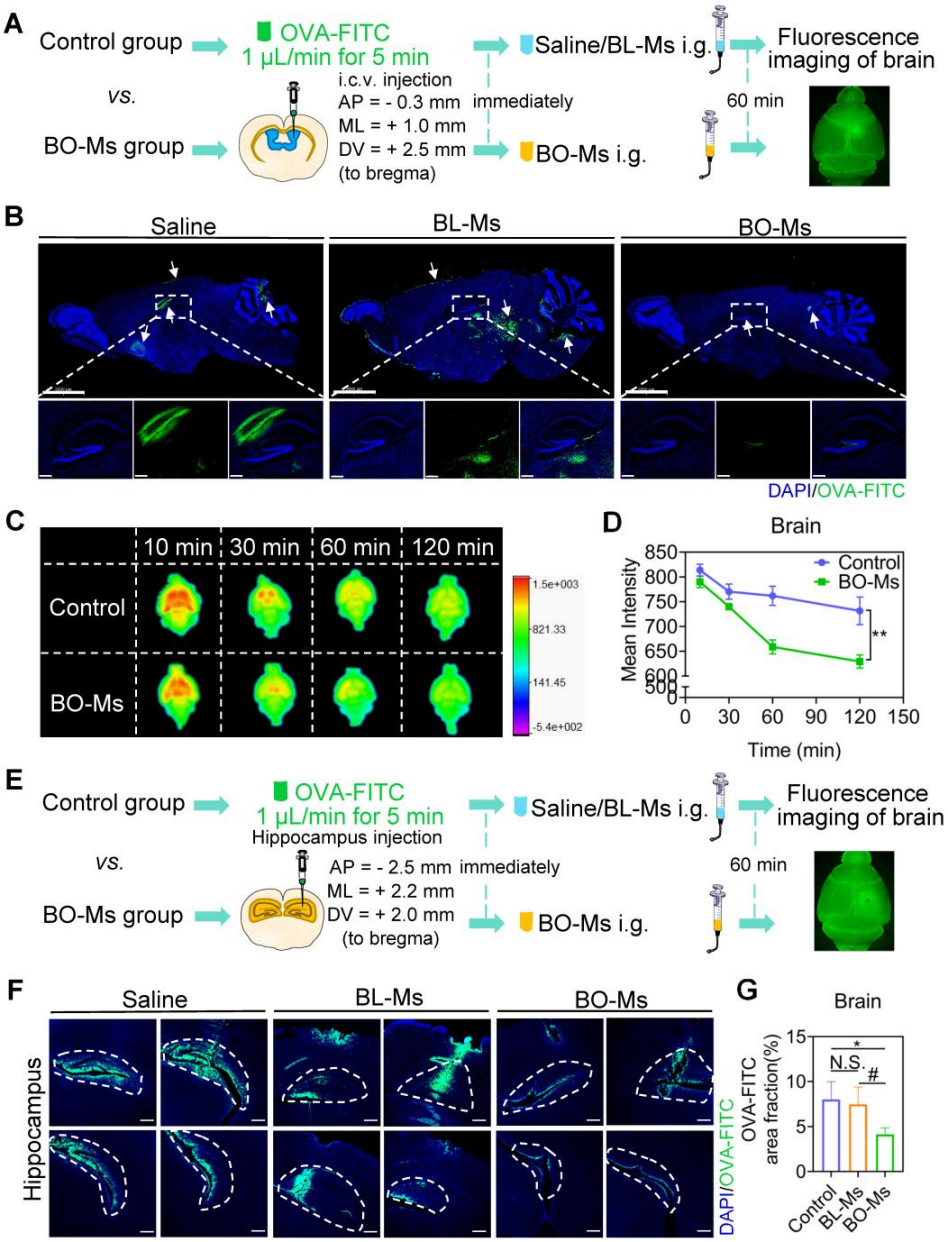
** BO-Ms improve the efflux of macromolecules from CNS. (A)** Schematic of the experimental layout where mice were injected intracerebroventricular (i.c.v.) with OVA-FITC and then intragastrical administration (i.g.) of BO-Ms. **(B)** Representative brain sections stained with DAPI (blue) showing OVA-FITC (green) distribution in the brain parenchyma of the control group, BL-Ms group, and BO-Ms group at 1 h. (Scale bars, 2 mm. White arrow: OVA-FITC). **(C)** The Ex vivo fluorescence imaging and **(D)** The intensity of OVA-FITC in mice brain in both groups at 10, 30, 60, and 120 min. **p < 0.01 (Student's *t*-test), n = 3 mice per group, representative of two independent experiments. Data are means ± SEM. **(E)** Schematic of the experimental brain was imaging when mice were administered intragastrically by the garage of saline or BO-Ms after OVA-FITC injected hippocampus. **(F)** Representative brain sections showing OVA-FITC in green and cell nuclei in blue. (Scale bars, 2 mm). **(G)** The coverage rate of OVA-FITC (percentage of brain section). *p < 0.05, ^#^*p* < 0.05, N.S. = not significant, (Student's *t*-test), n = 5 mice per group, representative of two independent experiments. Data are means ± SEM.

According to previous findings, borneol could quickly enter the brain within 5 min after oral administration 21. In this study, borneol was prepared into micelles for oral administration conveniently (Figure S1A). The transmission electron microscopy (TEM) revealed that BO-Ms were uniform and spherical particles (Figure S1B). The BO-Ms and BL-Ms showed hydrodynamic diameters of 20.06 ± 2.54 nm and 17.32 ± 1.49 nm, PDI of 0.291 ± 0.012 and 0.257 ± 0.050, and zeta potential of -2.45 ± 0.87 mV and -5.51 ± 0.83 mV, respectively (Figure S1C). To see if BO-Ms affected macromolecule clearance in the lateral ventricle, mice were given 20 mg/kg of BO-Ms orally after ovalbumin-FITC (OVA-FITC, 45 KD) injections into the lateral ventricle. Mice in the control group were given the same amount of normal saline and blank micelles (BL-Ms), respectively (Figure 1A). The signal of OVA-FITC that spread from the lateral ventricle into the hippocampus was significantly lower in the BO-Ms group than in the saline group and BL-MS group after 60 min administration (Figure 1B). Moreover, we found that the fluorescence of OVA-FITC in the brain decreased in both groups with time. However, as compared to the control group, the fluorescence value in the brain of the mice in the BO-Ms group was much lower (Figure 1C-D). To explore the scavenging function of BO-Ms on macromolecules in the brain, we employed micellar CY5.5 (CY5.5-Ms, ~2 KD) as another macromolecular model. CY5.5-Ms were observed as blue and with high fluorescence intensity by using an *in vivo* imaging system (Ex = 610 nm, Em = 700 nm) (Figure S2A-B). The average sizes of CY5.5-Ms were 15.12 ± 0.91 nm, and PDI was 0.205 ± 0.041 (Figure S2C). When CY5.5-Ms were injected into the lateral ventricle, the concentration of CY5.5-Ms in the brain of mice in the control group increased significantly quicker than in the BO-Ms group, and the AUC of the control group was 1.4-fold higher than that of BO-Ms group (Figure S3A-C). These results suggested that BO-Ms could promote macromolecule clearance in the CSF. The effect of BO-Ms on the efflux of macromolecules from the brain parenchyma was then explored further. After injecting OVA-FITC into the hippocampus, saline and BL-Ms or BO-Ms (20 mg/kg) were administered orally as a control group or BO-Ms group, respectively (Figure 1E). 60 min later, compared to the saline and BL-Ms group, treatment with BO-Ms resulted in a significant decrease in the trace coverage in the hippocampus (Figure 1F-G), indicating that BO-Ms also facilitate the macromolecule clearance in the brain parenchyma. In summary, BO-Ms played an important role in increasing the clearance of macromolecules in the CNS. Besides, there was no significant difference in the clearance of macromolecules in the brain between the saline group and the BL-Ms group, thus the follow-up experiments used saline orally as the control group.

### BO-Ms drain the macromolecules in the CNS via the meningeal lymphatic vessels to dCLNs

**Figure 2:**
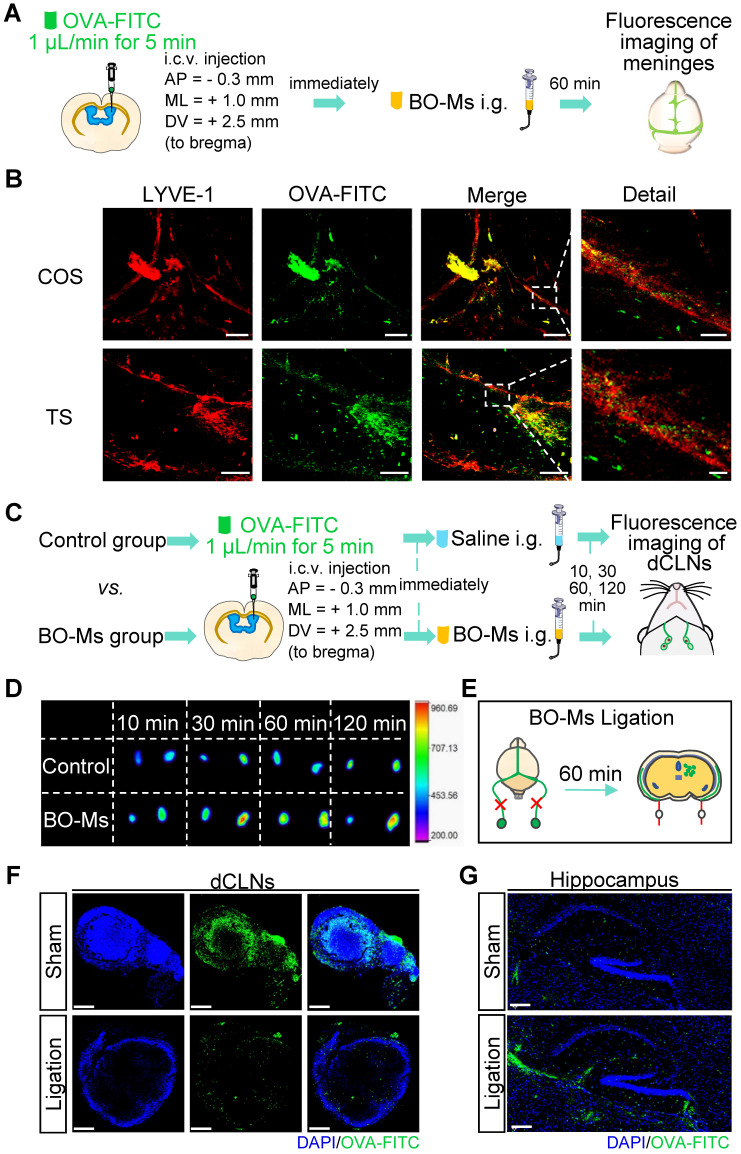
** BO-Ms clear tracers from the CNS into dCLNs via meningeal lymphatics. (A)** Schematic of the experimental layout where mice were injected intracerebroventricular (i.c.v.) with OVA-FITC and then intragastrical administration (i.g.) of normal saline as a control group or BO-Ms as BO-Ms group. The meninges were harvested at 60 min after administration. **(B)** Representative the confluence of sinuses (COS), and the transverse sinus (TS) of meninges stained with anti-LYVE-1 antibody (red) showing OVA-FITC (green) distribution in the meningeal lymphatic vessels of the control group and BO-Ms group at 60 min. (Scale bars in the COS, 200 μm, detail, 50 μm. Scale bars in the TS, 100 μm, detail, 20 μm). **(C)** Schematic of the experimental layout where mice were injected intracerebroventricular (i.c.v.) with OVA-FITC and then intragastrical administration (i.g.) of normal saline as a control group or BO-Ms as BO-Ms group. The dCLNs were harvested at various times after administration. **(D)** The Ex vivo fluorescence imaging of OVA-FITC in mice dCLNs in both groups at 10, 30, 60, and 120 min. **(E)** Schematic of the experimental ligation of the afferent lymphatic vessel of the dCLNs. **(F, G)** After afferent lymphatic vessel ligation, representative dCLNs and hippocampus in the BO-Ms sham group and BO-Ms ligation group showed OVA-FITC in green and cell nuclei in blue. (Scale bars in the dCLNs sections, 200 μm. Scale bars in the brain sections, 100 μm).

Meningeal lymphatic vessels have been confirmed to drain CSF and CNS-derived molecules from the brain into the dCLNs 35. To determine whether BO-Ms could drain the macromolecules released in the brain into dCLNs by the meningeal lymphatics, BO-Ms were given orally at the doses of 20 mg/kg after OVA-FITC was injected into the lateral ventricle of mice, and meninges were harvested 60 min later to assess for the presence of OVA-FITC (Figure 2A). Anti-LYVE-1 antibody was used to mark meningeal lymphatic vessels, which were then co-localized with the fluorescence of OVA-FITC. OVA-FITC was shown to collect around and inside the COS of the meninges, as illustrated in Figure 2B. Furthermore, the signal of OVA-FITC was shown to co-localize with LYVE-1-positive lymphatic endothelial cells in the meningeal TS (Figure 2B). This could be due to borneol enhancing macromolecules targeting the peripheral lymphatic system 30, whereas meningeal lymphatic vessels have similar lymphatic characteristics and express all molecular markers of lymphatic endothelial cells 36,37, allowing BO-Ms to increase the transport of macromolecular to the meningeal lymphatic system. Furthermore, lymphatic pathways flowing through the cribriform plate play a significant role in removing macromolecules from the brain and transporting them to the extracranial lymphatic system 38,39. To determine whether BO-Ms could enhance CSF flow through lymphatic drainage near the cribriform plate, mice were injected with EB into the cisterna magna, because EB is a high affinity for serum albumin to be preferentially drained via the lymphatics 40,41, then mice were orally administrated BO-Ms at the dosages of 20 mg/kg, while the control group were given the same amount of normal saline (Figure S4A). Afterwards, the injected heads were sliced along the midline for imaging (Figure S4B). As shown in Figure S4B, EB was observed in the nasal epithelium of the mice in both groups, and the color of the EB gradually deepened over time. Furthermore, as compared to the control group, the EB in the nasal epithelium of the mice in the BO-Ms group was more broadly distributed, indicating that BO-Ms accelerated the outflow of cerebrospinal fluid via the cribriform lymphatic pathway.

Tracers and proteins injected in the CNS are drained into dCLNs by meningeal lymphatics 14,35. To explore whether the effect of BO-Ms on the drainage function of meningeal lymphatic vessels that drain macromolecules released into the CSF into the dCLNs, we injected OVA-FITC and CY5.5-Ms into the lateral ventricle of mice, followed by the immediate administration of BO-Ms by gavage. The control group mice were given normal saline orally, and the dCLNs were harvested at different times to determine, respectively (Figure 2C, Figure S5A). The fluorescence intensity of OVA-FITC in dCLNs of the control and BO-Ms groups increased with time, with the BO-Ms group having a greater fluorescence intensity than the control group (Figure 2D). After BO-Ms therapy, the content of CY5.5 in the dCLNs may be gradually raised in comparison to the control group (Figure S5B). Simultaneously, the dCLNs were seen to be visibly blue and CY5.5 tagged 1 h after BO-Ms oral gavage (Figure S5C). However, when the afferent lymphatic vessels were ligated (Figure 2E, Figure S5D), the OVA-FITC in the dCLNs in the BO-Ms ligation group was less than that in the BO-Ms sham group (Figure 2F). Compared with the BO-Ms sham group, the dCLNs in the BO-Ms ligation group did not seem blue marked by CY5.5-Ms, and CY5.5-Ms concentration was also significantly decreased (Figure S5E-F). In addition, the fluorescence level of OVA-FITC in the hippocampus of the BO-Ms ligation group was higher than that of the BO-Ms sham group (Figure 2G). These findings indicated that BO-Ms might drain macromolecules from CNS into dCLNs through meningeal lymphatic vessels.

### BO-Ms promote meningeal lymphatic drainage

**Figure 3:**
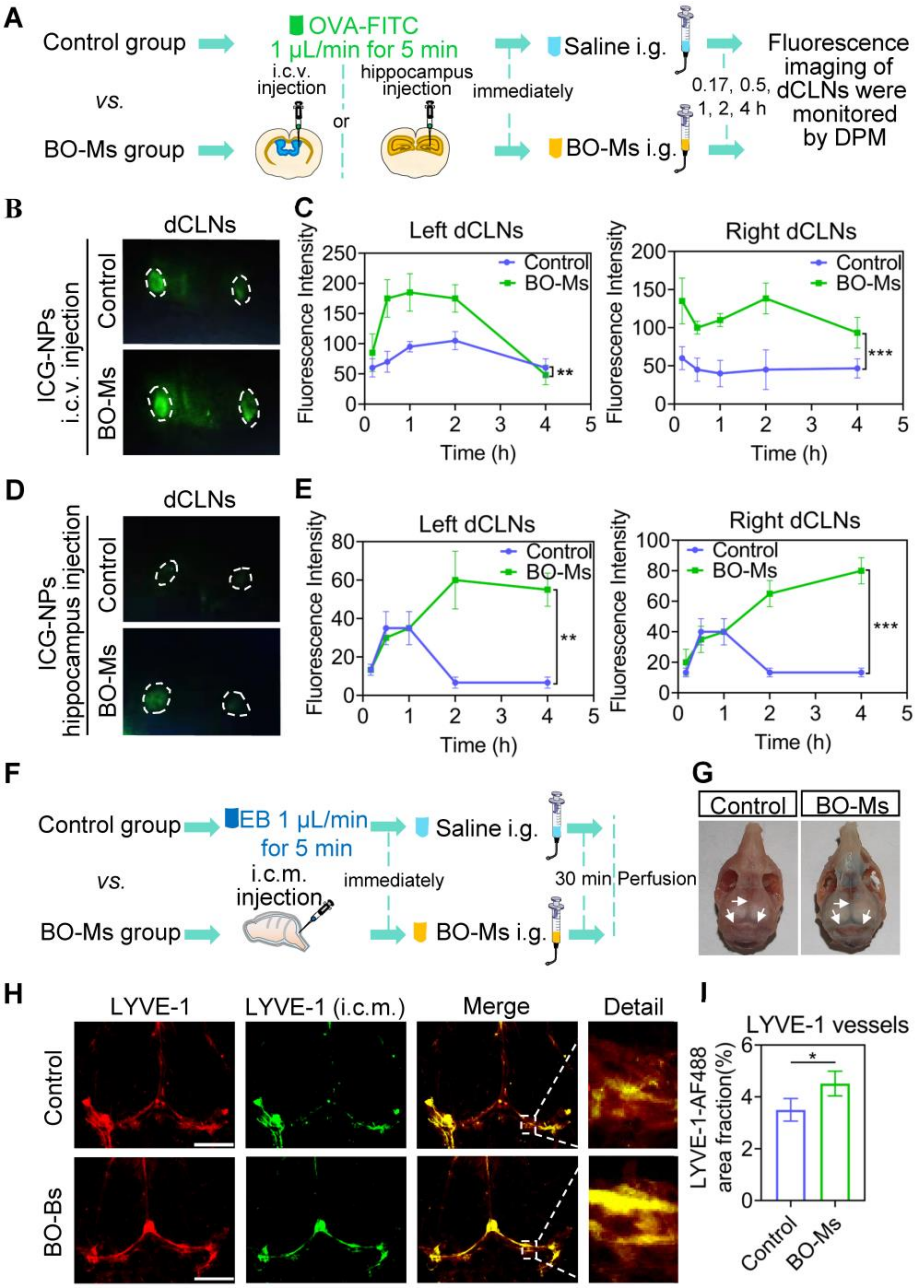
** BO-Ms increase drainage efficiency of meningeal lymphatic for uptake of cerebrospinal fluid. (A)** Schematic of the experimental layout where mice were injected ICG-NPs and then intragastrical administration (i.g.) of saline or BO-Ms, the dCLNs were monitored at different time points by the near-infrared fluorescence imaging system of DPM.** (B)** Representative fluorescence imaging and **(C)** intensity of ICG-NPs in mice left and right dCLNs in both groups after ICG-NPs injected in lateral ventricle. **p < 0.01, ***p < 0.001 (Student's *t*-test), n = 3 mice per group, representative of two independent experiments. Data are means ± SEM. **(D)** Representative fluorescence imaging and **(E)** intensity of ICG-NPs in left and right dCLNs in control and BO-Ms groups after ICG-NPs injected in the hippocampus. **p < 0.01, ***p < 0.001 (Student's *t*-test), n = 3 mice per group, representative of two independent experiments. Data are means ± SEM. **(F)** Schematic of the experiments that mice were injected in intra-cisterna magna (i.c.m.) with EB, and then the saline or BO-Ms was orally administered in order to measure drainage into meningeal lymphatic vessels. **(G)** Distribution of EB in meningeal lymphatics in control and BO-Ms group. White narrows: EB. **(H)** Representative meninges stained with anti-LYVE-1 (CY3-conjugated secondary antibody, immunofluorescence) and AF488-conjugated anti-LYVE-1 (i.c.m.) distribution in the meningeal lymphatic vessels of the control group and BO-Ms group at 30 min. (Scale bars, 3 mm). **(I)** The coverage rate of AF488-conjugated anti-LYVE-1 antibody (percentage of meninges). *p < 0.05 (one-way ANOVA with Student's *t*-test), n = 3 mice per tissue, representative of two independent experiments. Data are means ± SEM.

To certify that BO-Ms indeed enhanced meningeal lymphatic drainage efficiency in clearing macromolecules from the brain, we injected indocyanine green nanoparticles (ICG-NPs) into the lateral ventricle and hippocampus, followed by 20 mg/kg BO-Ms given orally immediately, while the control group given the same amount of normal saline orally. ICG-NPs were green macromolecular complexes with an average size of 201.93 ± 4.52 nm that allow for *in vivo* real-time fluorescence imaging (Figure S6A-C). The fluorescent flow of ICG-NPs in the dCLNs was then traced by a near-infrared fluorescence imaging system of DPM at 0.17, 0.5, 1, 2, and 4 h, respectively (Figure 3A). When ICG-NPs were injected into the lateral ventricle, the fluorescence of ICG-NPs filled in dCLNs in both groups at 0.17 h, whereas the fluorescence intensity of the BO-Ms group being higher than that of the control group (Figure 3B-C). The Tmax values in the left dCLNs of the BO-Ms group were shorter (1 h) than in the control group (2 h), implying BO-Ms can quickly improve the clearance rate of macromolecules in the CSF (Figure 3C). Within 4 h, the area under the curve (AUC) of ICG-NPs was greater in the BO-Ms group than in the control group, indicating that BO-Ms increased the drainage quantity of meningeal lymphatic vessels to macromolecules in the brain (Figure 3C). Besides, BO-Ms could promote the transport of ICG-NPs injected in the hippocampus into dCLNs at 0.17 h after BO-Ms oral administration, with more fluorescence observed in the BO-Ms group's dCLNs compared to the control group (Figure 3D-E). The Cmax and AUC of ICG-NPs were significantly higher than in the BO-Ms group than in the control group (Figure 3E), illustrating that BO-Ms significantly increased the degree of macromolecular clearance in brain parenchyma.

The removal of macromolecules in the brain is mainly through the meningeal lymphatic pathway 35. To explore whether BO-Ms affect the function of meningeal lymphatic vessels, which assisted in the clearance of macromolecules in the brain, the EB was injected in the cisterna magna, and then the BO-Ms were administrated to mice via oral gavage at 20 mg/kg, while control group mice were given the same dosages of normal saline (Figure 3F). After 30 min of oral delivery, the EB in the BO-Ms group was widely distributed in the SSS, TS, and COS of meninges in the skull, as shown in Figure 3G, while the EB in the control group was not clearly detected. Furthermore, we injected AF488-conjugated anti-LYVE-1 in the intra-cisterna magna, and the percentage of meningeal lymphatics labeled by AF488-anti-LYVE-1 antibody (i.c.m.) divided by the area of meningeal lymphatics represented the speed of meningeal lymphatic flow 13. Following an AF488-conjugated anti-LYVE-1 injection, the mice were given either saline or BO-Ms groups, respectively. The fluorescent via dorsal meningeal lymphatic drainage route was monitored at 30 min after injection and the meninges were harvested and stained for LYVE-1 using a CY3-conjugated secondary antibody. BO-Ms group mice had significantly higher fluorescence intensity and area in the meninges surface than the control group mice (Figure 3H-I). BO-Ms increased tracer drainage via meningeal lymphatics when compared with those in the control group (Figure 3H-I), which may be related to BO-Ms promoting CSF uptake by meningeal lymphatics.

### BO-Ms enhance meningeal lymphatic drainage depending on the lymphangiogenesis and lymphatic flow

**Figure 4:**
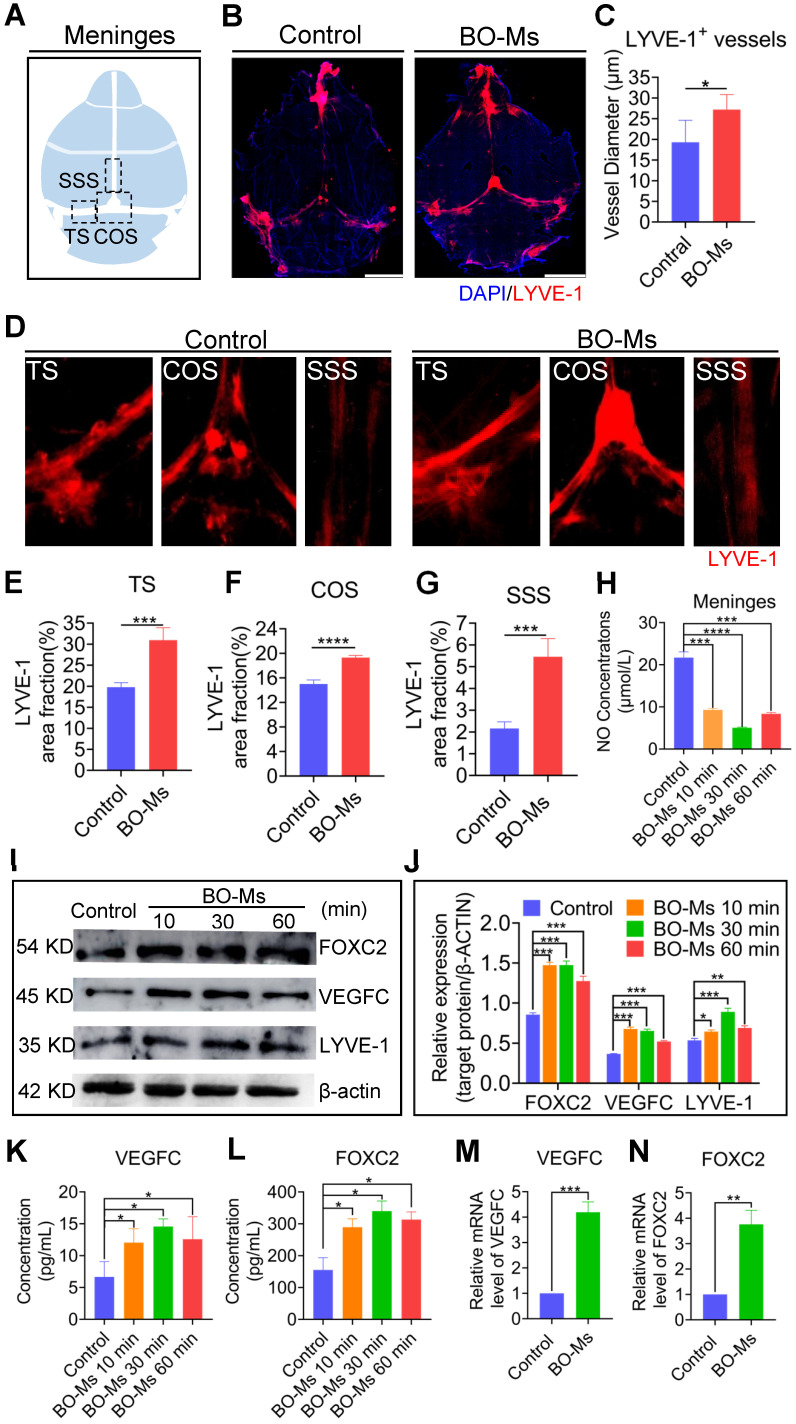
** Effect of BO-Ms on the changes in meningeal lymphatic vessels morphology and the expression of lymphoid markers in the meninges. (A)** Schematic of the meninges. TS: transverse sinus, COS: confluence of sinuses, SSS: superior sagittal sinus. **(B)** Representative image of meninges stained with DAPI (blue) anti-LYVE-1 antibody (red). (Scale bars, 3 mm) **(C)** Quantification of the diameters of the meningeal lymphatic vessels. *p < 0.05 (one-way ANOVA with Student's *t*-test), n = 3 mice per tissue, representative of two independent experiments. Data are means ± SEM. **(D)** Representative image of meninges stained with anti-LYVE-1 antibody (red) in TS, COS, and SSS. **(E-G)** Quantification of the percent area coverage of LYVE-1 antibody staining in the TS, COS, and SSS of meninges. ***p < 0.001, ****p < 0.001 (one-way ANOVA with Student's *t-*test), n = 3 mice per tissue, representative of two independent experiments. Data are means ± SEM. **(H)** The concentration of NO of meninges in BO-Ms oral groups and control group at different times. ***p < 0.001, ****p < 0.001 (Student's *t*-test), n = 3-4 mice per tissue, representative of two independent experiments. Data are means ± SEM. **(I)** Representative western-blot images of LYVE-1, FOXC2, and VEGFC in the meninges of mice in the control and BO-Ms groups at 10, 30, and 60 min. β-actin was used as an internal reference protein. **(J)** Quantification of LYVE-1, FOXC2, and VEGFC expression. *p < 0.05, **p < 0.01, ***p < 0.001 (Student's *t*-test), n = 3 mice per tissue, representative of two independent experiments. Data are means ± SEM. (K-L) Content of VEGFC and FOXC2 in the meninges measured by ELISA. *p < 0.05 (Student's *t*-test), n = 3 mice per group. Data are means ± SEM. (M-N) mRNA expression levels of VEGFC and FOXC2 in meninges. **p < 0.01, ***p < 0.001 (Student's* t*-test), n = 3 mice per tissue. Data are means ± SEM.

The morphological alterations of meningeal lymphatic vessels were explored initially to investigate the mechanism of BO-Ms on augmentation of meningeal lymphatic functions to improve the drainage efficiency of macromolecules in the brain. After 30 min of BO-Ms oral gavage at a dosage of 20 mg/kg, the meningeal lymphatic vessels were harvested and labeled with the anti-LYVE-1 antibody (Figure 4A-B). The lymphatic diameter was also quantified to assess changes in LYVE-1 structure. The lymphatic diameter in the BO-Ms gavage group was significantly greater than in the control group (Figure 4C). As shown in Figure 4D-G, after treatment with BO-Ms, the LYVE-1-positive area in meningeal lymphatic vessels was increased, and the percent area coverage of LYVE-1 by the area of meningeal whole-mounts in SSS (2.5-fold), COS (1.2-fold) and TS (1.6-fold) in the BO-Ms group were generally higher than that in the control group. Since the meningeal lymphatic vessels express all of the molecular features of the lymphatic endothelium, the dilating effect of borneol on the lymphatic vessels in the legs was further verified 42. Figure S7A showed that the inner diameter of the lymphatic vessels in the legs of the BO-Ms group expanded 2-fold when compared to the control group.

Lymphatic contraction is the dynamic basis of lymphatic circulation and is critical in maintaining the steady state of the circulatory system. NO is a key regulator of lymphatic pumping and is essential for lymphatic regulation. Under physiological settings, the periodic variations of the bioactive molecule NO are implicated in the regulation of lymphatic channel contraction, relaxation, and tension 43. Therefore, the NO of meninges was measured after intragastrical administration of BO-Ms (20 mg/kg), and a lower concentration of NO of meninges was detected in the BO-Ms group compared to the control group (Figure 4H), suggesting that BO-Ms could improve lymphatic vessel contractility and thus increase lymphatic circulation. Studies have shown that the expression of VEGFC, FOXC2, and LYVE-1 plays an important role in the lymphangiogenesis and maturation of lymphatic vessels. VEGFC increases the generation of lymphatic endothelial cells, which then migrate to constitute primary lymphatic vessels. FOXC2 is a key marker in the maturation of primary lymphatic vessels, allowing them to perform normal functions. LYVE-1, a lymphatic endothelial cell marker, correlates with the number of lymphatic endothelial cells quantity 44-47. Western blot results showed that the expression of lymphangiogenesis markers such as VEGFC, FOXC2, and LYVE-1 was remarkable in the BO-Ms group during 2 h (Figure 4I-J). The ELISA and RT-qPCR results revealed that after treatment with BO-Ms, the protein and the mRNA expression of VEGFC and FOXC2 in the meninges increased significantly (Figure 4K-N). Because lymphatic endothelial cell proliferation and migration are important in lymphangiogenesis 48, the effect of borneol on lymphatic endothelial cell proliferation and migration was evaluated. The borneol improved the cell proliferation ability of MLECs, as shown in Figure S8A. In terms of migration assessment, borneol has the potential to significantly increase the migration of MLECs from the upper to the lower chamber (Figure S8B). As a result, BO-Ms may enhance meningeal lymphatic drainage ability by improving the morphology, production, and function of meningeal lymphatic endotheliocytes, suggesting that BO-Ms promoted clearance of macromolecules by the meningeal lymphatic flow.

### BO-Ms increase drainage of Aβ_42_ oligomers from brain into dCLNs via meningeal lymphatic vessels

**Figure 5:**
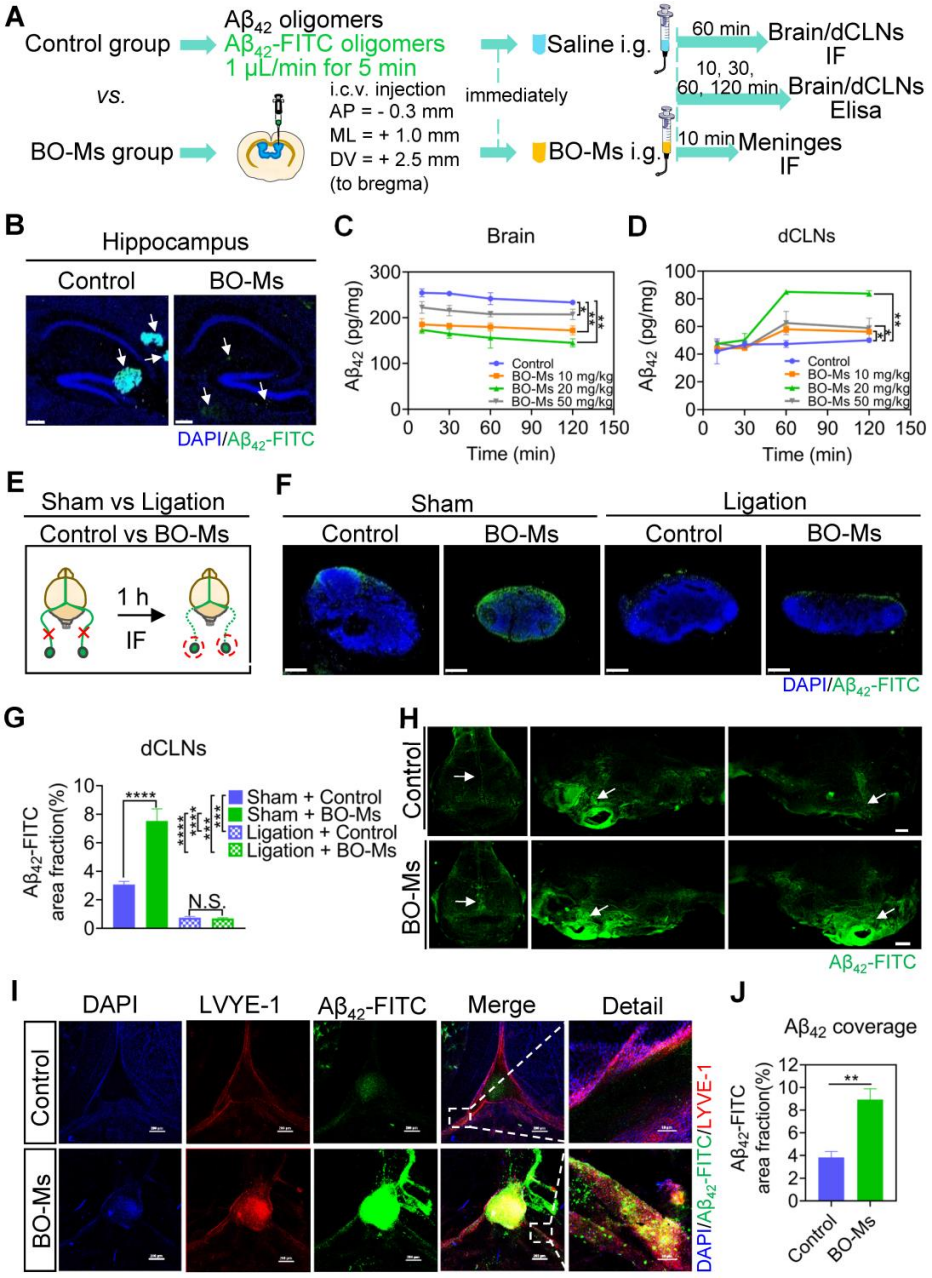
** BO-Ms clear Aβ_42_ oligomers from the brain into dCLNs via meningeal lymphatic vessels. (A)** Schematic of the experimental layout where mice were injected intracerebroventricular (i.c.v.) with Aβ_42_ oligomers or Aβ_42_-FITC oligomers and then intragastrical administration (i.g.) of saline or BO-Ms. The brain, meninges and dCLNs of mice in both groups were harvested at different time points.** (B)** Representative brain sections stained with DAPI (blue) showing Aβ_42_-FITC oligomers (green) distribution in the hippocampus in the control group and BO-Ms group at 60 min. (Brain parenchyma scale bars, 500 μm, hippocampus scale bars, 200 μm). White arrow: Aβ_42_-FITC oligomers. **(C)** Content of Aβ_42_ oligomers in the brains measured by ELISA. **p < 0.01 (Student's *t*-test), n = 3 mice per group, representative of two independent experiments. Data are means ± SEM. **(D)** Content of Aβ_42_ in the dCLNs measured by ELISA. **p < 0.01 (Student's *t-*test), n = 3 mice per group, representative of two independent experiments. Data are means ± SEM. **(E)** Schematic of the experimental ligation of the afferent lymphatic vessel of the dCLNs in the control group mice and BO-Ms group mice. **(F)** Representative dCLNs sections showing Aβ_42_-FITC oligomers in green and cell nuclei in blue after afferent lymphatic vessel ligation. (Scale bars, 200 μm). **(G)** The coverage rate of Aβ_42_-FITC oligomers (percentage of brain section) in the dCLNs in the sham group and ligation groups with or without BO-Ms treatment. N.S. = not significant, ***p < 0.001, ****p < 0.001 (Student's *t*-test), n = 3 mice per group, representative of two independent experiments. Data are means ± SEM. **(H)** Distribution of Aβ_42_-FITC oligomers in meninges. White arrow: Aβ_42_-FITC oligomers. (Scale bars, 2 mm). **(I)** Representative meninges stained with DAPI (blue) and anti-LYVE-1 antibody (red) showed Aβ_42_-FITC oligomers (green) distribution in the meningeal lymphatic vessels of the control group and BO-Ms group at 10 min. (Scale bars, 200 μm, details figures scale bars, 50 μm). **(J)** The coverage rate of Aβ_42_-FITC oligomers (percentage of meninges). **p < 0.01 (Student's *t*-test), n = 3 mice per group, representative of two independent experiments. Data are means ± SEM.

Aβ is a toxic amyloid protein in the brain, and the aggregation, caused by the imbalance in its production and clearance, is a key factor in the pathogenesis of AD. Therefore, effective Aβ removal in the brain is key to preventing and treating AD 49. We demonstrated that BO-Ms can enhance of meningeal lymphatic drainage to remove macromolecules from the brain. To see if BO-Ms could promote Aβ clearance in the brain via meningeal lymphatic vessels, we injected Aβ_42_-FITC oligomers into the lateral ventricle of the mice, and then immediately administered BO-Ms orally, while control group mice were given the same amount of normal saline (Figure 5A). The distribution of Aβ_42_-FITC oligomers in the hippocampus was observed 60 min after oral administration (Figure 5A-B). As shown in Figure 5B, the control group had an aggregation of Aβ_42_-FITC oligomers in the hippocampus, whereas the BO-Ms group had a scattered distribution of Aβ_42_-FITC oligomers in the hippocampus. Besides, the quantity of Aβ_42_-FITC oligomers in the BO-Ms group was lower than in the control group, suggesting BO-Ms facilitated clearance of Aβ_42_-FITC oligomers in the CNS (Figure 5B). The content of Aβ_42_ oligomers in the brain was significantly reduced after 10 min of BO-Ms treatment at doses of 10 mg/kg, 20 mg/kg, and 50 mg/kg when compared to the control group, indicating that BO-Ms improved the clearance rate of Aβ_42_ oligomers in the brain (Figure 5C). The area under the curve of Aβ_42_ content was significantly lower in the brain of the 10 mg/kg, 20 mg/kg, and 50 mg/kg BO-Ms groups (19927.25 mg/L*h, 17823.70 mg/L*h, and 23747.42 mg/L*h) than in the control group (27429.83 mg/L*h), indicating that BO-Ms increased the clearance amount of Aβ_42_ content in the brain (Figure 5C). The content of Aβ_42_ oligomers in the dCLNs of the control and BO-Ms groups increased with time, and at 60 min, the content of Aβ_42_ oligomers in the dCLNs of the BO-Ms group was significantly higher than that of the control group (Figure 5D). The area under the curve of Aβ_42_ content in the dCLNs of the 10 mg/kg, 20 mg/kg, and 50 mg/kg BO-Ms groups was higher 1.12-, 1.54-, and 1.18-fold than that of the control group, respectively (Figure 5D). However, the drainage effect of BO-Ms was impaired as the afferent lymphatic vessels of the dCLNs were ligated (Figure 5E). When compared to the sham-operated group, the distribution of Aβ_42_-FITC oligomers in the dCLNs was significantly decreased after the afferent lymphatic vessels were ligated of the mice in the control ligation group and the BO-Ms ligation group, and there was no difference in the distribution of Aβ_42_-FITC oligomers in the dCLNs between control ligation group and BO-Ms ligation group (Figure 5F-G). In summary, these results indicated that BO-Ms promote the clearance of Aβ_42_ oligomers in the brain into the dCLNs through lymphatic pathway.

A growing body of evidence indicates that meningeal lymphatics are responsible for Aβ clearance in aged mice and transgenic mouse models of AD 8. In order to investigate the effect of BO-Ms on meningeal lymphatic vessels to clear Aβ, Aβ_42_-FITC oligomers were injected into the lateral ventricle of the mice, followed by an oral administration of BO-Ms, while control group mice were given the same amount of normal saline (Figure 5A). The meninges were harvested 10 min later, and the meningeal lymphatic endothelial cells were labeled with anti-LYVE-1 antibodies before colocalization with Aβ_42_-FITC oligomers (Figure 5I). As shown in Figure 5I, the Aβ_42_-FITC oligomers in the BO-Ms group filled in the inside and around the meningeal lymphatic vessels, whereas the saline-treated group showed less distribution of Aβ_42_-FITC oligomers in the meningeal lymphatic vessels. Likewise, we discovered an increase in Aβ_42_-FITC oligomers in meningeal lymphatic vasculature after BO-Ms treatment (Figure 5J). Moreover, more fluorescence of Aβ_42_-FITC oligomers was observed in the basal meningeal lymphatics via BO-Ms oral gavage (Figure 5H). Previous studies reported that basal meningeal lymphatics were the hotspots for the clearance of CSF macromolecules 46. In addition, we injected Aβ_42_-FITC into the lateral ventricles, and the mice were immediately given saline or BO-Ms orally as a control and BO-Ms group, respectively (Figure S9A). The cribriform plate tissues were harvested after 30 min and stained for LYVE-1 with a CY3-conjugated secondary antibody. The fluorescence of Aβ_42_-FITC could co-localize with the lymphatic vessels labeled with anti-LYVE-1 antibody. BO-Ms group mice had significantly higher LYVE-1 fluorescence intensity and area in the cribriform plate than the control group mice (Figure S9B-C). These findings indicated that BO-Ms facilitated the transport of Aβ from CNS into the lymphatic vessels in the meninges and nasal epithelium, where it was then drained into dCLNs.

### BO-Ms alleviate the Aβ burden and ameliorate learning and memory deficits in mouse models of AD

**Figure 6:**
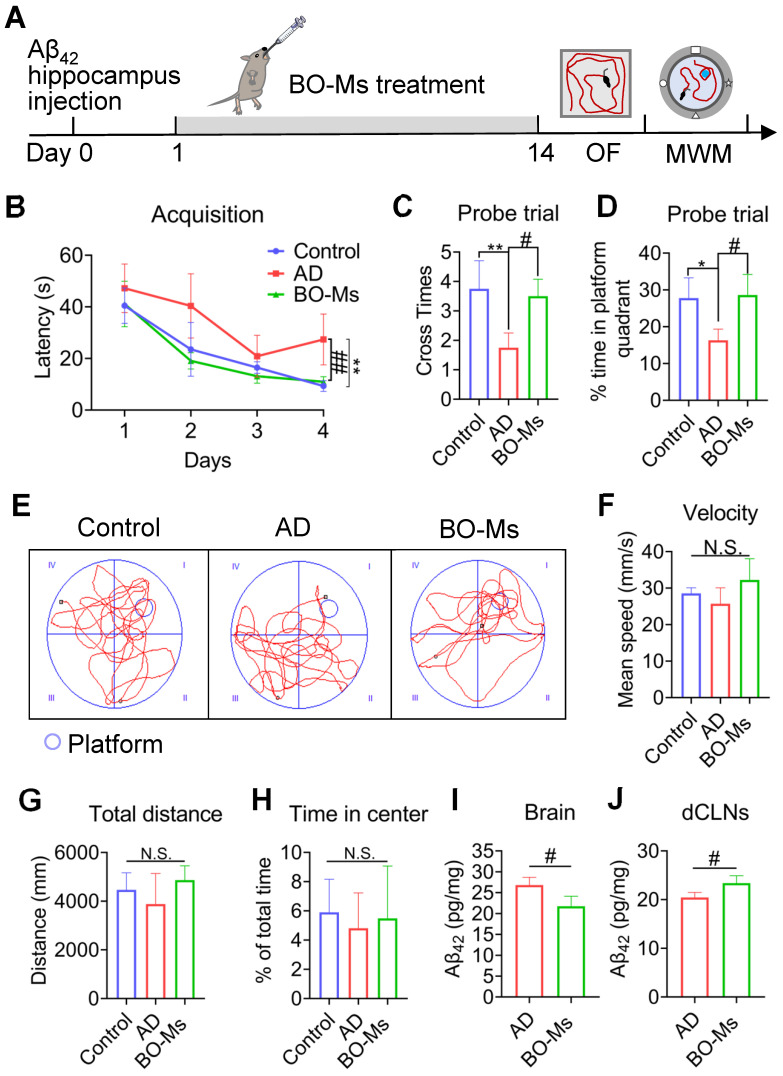
** BO-Ms improved memory deficits in AD mice. (A)** Timeline of BO-Ms treatment in AD mice. **(B)** Escape latency to the platform during the training trails in a Morris water maze.^ **^p < 0.01, *vs.* control group. *^##^p* < 0.01, *vs.* AD group. n = 6 mice per group. Data are means ± SEM. **(C)** The number of target platform crossings in the probe test. ^**^p < 0.01, *vs.* control group. n = 6 mice per group. *^#^p* < 0.05, *vs.* AD group. n = 6 mice per group. Data are means ± SEM. **(D)** Time spent in the target quadrant in the probe test. ^*^p < 0.05, *vs.* control group. n = 6 mice per group. *^#^p* < 0.05, *vs.* AD group. n = 6 mice per group. Data are means ± SEM. **(E)** Representative track images of mice in the probe test. **(F)** Mean swimming velocity of mice. N.S. = not significant. **(G)** Total distance in open field arena. N.S. = not significant. **(H)** Percentage of time in the center of the open field arena. N.S. = not significant.** (I)** Content of Aβ_42_ oligomers in the brains measured by ELISA. *^#^p* < 0.05, *vs.* AD group. n = 3 mice per group. Data are means ± SEM. **(J)** Content of Aβ_42_ in the dCLNs measured by ELISA. *^#^p* < 0.05, *vs.* AD group. n = 3 mice per group. Data are means ± SEM.

The Aβ_42_-induced AD mice model was used to examine the biological effects of BO-Ms and determine how well they treated cognitive impairment. An important element influencing the development and prognosis of AD is the possibility that Aβ may cause the death of nerve cells and the development of senile plaques in the brain 50. Aβ_42_ injected in bilateral intra-hippocampal resulting in both substantial neuronal loss and memory deficits 34. The model mice were given BO-Ms orally for 14 d with BO-Ms before the OF and MWM tests, which measured their spatial cognition and memory (Figure 6A). As seen by longer escape latency times, fewer platform crossings, and shorter time spent in the platform quadrant during the probing trial, AD mice had glaring deficiencies in spatial learning and memory as compared to control mice (Figure 6B-D). By receiving BO-Ms therapy, AD mice significantly reversed these deficits. Specifically, mice treated with BO-Ms exhibited lower escape latencies, more platform crossing, and longer swimming time at the target location (Figure 6B-D). In addition, each group's mouse swimming traces were distributed among the four quadrants. Nevertheless, the mice's swimming traces in the BO-Ms group were mostly found near the platform's target region (Figure 6E). The alleviation of these phenotypes after BO-Ms treatment was independent of swimming speed in mice (Figure 6F). In the OF test, there were no differences between the control group, the AD group, and the BO-Ms group in terms of total distance or central zone time (Figure 6G-H). Additionally, as compared to mice in the AD group, BO-Ms-treated animals showed a decrease in Aβ_42_ oligomer concentration in the brain (Figure 6I) and an increase in Aβ_42_ oligomer concentration in decline (Figure 6J). In summary, BO-Ms treatment dramatically ameliorated the spatial learning and memory deficits in AD mice, which may be attributed to the enhanced lymphatic clearance of Aβ_42_ oligomers in the brain into the dCLNs by BO-Ms.

Moreover, the Aβ monomers produced in the brain will gradually aggregate into higher-order oligomers and fibrils, which are detected in AD brain. This will cause cell function damage, such as decreased synaptic activity and synaptic loss, as well as impaired brain capillary blood flow 51. Therefore, the anti-dementia impact of BO-Ms on easing cognitive impairment was investigated using the Aβ_42_ fibrils generated AD mice model. After BO-Ms therapy for 14 d, mice were trained for the MWM task (Figure S10A). The escape latency for each group gradually decreased during the training test. Lower escape latency in mice given BO-Ms treatment than in the AD group suggested that BO-Ms effectively enhanced spatial learning (Figure S10B). In the spatial probe trails with the platform removed, the crossed time of the platform position and the time spent in the target quadrant in the BO-Ms group were higher than those in the AD group, suggesting BO-Ms effectively improved memory impairment in AD mice (Figure S10C-D). As shown in Figure S10E, compared to the AD group, the swimming traces of the BO-Ms group were mainly located in the target area where the platform had been located. However, there was no significant difference in the swimming speed of the mice in each group (Figure S10F). The BO-Ms group presented fewer Aβ plaque deposits in the hippocampus than the AD group, suggesting that BO-Ms significantly decreased Aβ deposition in the AD brain (Figure S10G). In conclusion, the aforementioned results showed that BO-Ms efficiently ameliorated cognitive deficits in Aβ_42_-induced AD model mice and improved the anti-dementia effect.

## Discussion

Previous studies have shown that BO-Ms could promote other components into the brain by crossing the blood-brain barrier (BBB), improving the therapeutic effect of brain diseases 52,53. In addition, there were reports also showing that BO-Ms regulated the BBB in both directions, which not only promoting the entry of other substances into the brain but also the efflux of substances in the brain. Meningeal lymphatics have recently been implicated in the outflow of macromolecules from the CNS 54. We previously demonstrated that borneol enhances macromolecule lymphatic targeting, increasing lymph node uptake 30. However, it is unknown whether BO-Ms can promote the efflux of components in the brain through meningeal lymphatic vessels. In this study, we identified a novel role of BO-Ms in facilitating the drainage of macromolecules from inside to the outside of the brain by modulating meningeal lymphatic vessels. When macromolecular tracers were injected into the brain of mice, oral administration of BO-Ms could accelerate the efflux of macromolecules to the dCLNs, but ligation of afferent lymphatic vessels of dCLNs would block the clearance effect of BO-Ms.

Meningeal lymphatic vessels are functional classic vasculature that may drain macromolecules to maintain CNS health while also being involved in a variety of neuropathological events 14. Meningeal lymphatic dysfunction leads to the imbalance of brain homeostasis, which worsens a range of neurodegenerative illnesses, including AD 8,13. Previous studies reported that viral delivery of VEGFC or intracerebroventricular injection of AAV1-GFP-DSCR1 was able to increase the diameter of meningeal lymphatic vessels to enhance meningeal lymphatic function 8,43, which were mainly based on molecular treatment regulation, with fewer small molecular compounds used. Our findings were novel in that oral treatment of BO-Ms raised lymphatic channel diameters and enhanced lymphatic vessel permeability to drain macromolecules delivered to the lymphatic circulation. BO-Ms also affected the concentration of NO, a lymphatic pump regulator, stimulated lymphatic vessel contraction, and accelerated lymphatic circulation. Besides, BO-Ms up-regulated the expression of lymphatic endothelial markers (VEGFC, FOXC2, and LYVE-1) of meningeal lymphatic vessels, indicating that BO-Ms effectively enhanced macromolecule clearance in the brain by promoting lymphangiogenesis and lymphatic vessels maturation. However, further research should be undertaken to investigate the regulatory effect of BO-Ms on meningeal lymphatic vessels in different physiological states, as well as the molecular mechanism by which borneol enhances the function of meningeal lymphatic vessels.

Our findings presented in this paper suggest that BO-Ms facilitated the uptake and clearance of CSF by the meningeal lymphatic system. In addition, in the dorsal and basal meningeal areas, the tracer fluorescence in the BO-Ms group was greater than that in the control group. Recent studies have shown that hotspots in the dorsal and basal meningeal systems may be areas where cerebrospinal fluid is easy to enter 8,14,55. Nevertheless, it is yet unknown how BO-Ms increased CSF absorbed by meningeal lymphatic channels and removed from the CNS.

Increasing evidence points to an imbalance between Aβ production and clearance as the cause of Aβ buildup and aggregation in the brain 45. Accumulating evidence has shown that the meningeal lymphatic system and Aβ clearance are closely related processes. Aβ accumulation may induce lymphatic dysfunction and give rise to a positive feedback loop that decreases Aβ clearance 7-9. Besides, a recent study verified that the microglial inflammatory response in AD is caused by decreased meningeal lymphatic drainage and that enhancing meningeal lymphatics in conjunction with immunotherapies will increase Aβ clearance 19. Our research innovatively found that BO-Ms increased the effectiveness of Aβ clearance from the brain into the periphery via meningeal lymphatic vessels. These results indicated that the meningeal lymphatic system's ability to remove the Aβ was greatly influenced by BO-Ms. However, little is known about the mechanisms by which BO-Ms scavenge Aβ through other mechanisms, such as the glymphatic system and microglia-mediated clearance. Therefore, determining how much Aβ was removed by BO-Ms through the meningeal lymphatic pathway would be very important.

A large number of studies have shown that acute exposure of oligomeric Aβ in the hippocampus of rats leads to spatial learning and memory impairment, neuronal loss and positive amyloid deposition 56,57. These behavioral deficits are due to a transient increase in Aβ, which could characterize the early stage of AD pathogenesis 58. This study aimed to examine the therapy effect of BO-Ms on acute exposure of Aβ in the mice. Thus, oligomeric Aβ_42_-injected mice are a great model in the assessment of BO-Ms contribution to Aβ clearance in the* in vivo* brain. To achieve this, mice were injected bilaterally with Aβ_42_ oligomers and fibrils into the hippocampus. Our results revealed that borneol could improve the learning and memory deficits and reduce the amyloid deposits in the Aβ_42_-injected mice, which suggesting that borneol had a therapeutic effect on the early stages of AD.

In conclusion, the findings of this study emphasized the value of BO-Ms in improving meningeal lymphatic drainage. The meningeal lymphatic network, a clearance pathway, is involved in the process of various nervous system diseases, including AD, aging, and Parkinson's disease. BO-Ms augmented the meningeal lymphatic drainage and increased the clearance of macromolecules from the brain. The pharmacology of BO-Ms should thus be further studied since it might be used to prevent or cure neurological illnesses linked to aging and meningeal lymphatic system abnormalities.

## Abbreviations

Aβ: amyloid-beta peptide
AD: Alzheimer's disease
AChE: acetylcholinesterase
AUC: area under the curve
BBB: blood-brain barrier
BO-Ms: borneol micelles
CLN_S_: cervical lymph nodes
CNS: central nervous system
COS: confluence of sinuses
CSF: cerebrospinal fluid
CY5.5-Ms: CY5.5 Micelles
dCLNs: deep cervical lymph nodes
EB: even blue
HA: hyaluronic acid
ICG-NPs: ICG nanoparticles
ICG: Indocyanine green
ICG-NIR: Indocyanine green near-infrared
i.c.v.: lateral ventricle injection
i.c.m: Intra-cisterna magna injections
MWM: Morris water maze
NO: Nitric Oxide
OA: octadecylamine
OF: Open field
OVA-FITC: Ovalbumin-FITC
PFA: paraformaldehyde
SSS: superior sagittal sinus
TS: transverse sinus
TEM: transmission electron microscopy
VEGFC: vascular endothelial growth factor C
