# Supplementary material for: Borneol-driven meningeal lymphatic drainage clears amyloid-β peptide to attenuate Alzheimer-like phenotype in mice
Source: Theranostics. 2023 Jan 1;13(1):106–24. doi: 10.7150/thno.76133 (PMC9800736; doi:10.7150/thno.76133)
Supplement: Supplementary file 1 — Supplementary figures and table. [file thnov13p0106s1.pdf]

Supplementary Material

Table S1. Primer sequences and conditions for RT-qPCR.

Target	Product length (bp)	Annealing temperature (°C)	Primer (5’-3’)	Genbank accession no.
R-VEGFC-S	147	60	AACCCCTGAATCCTGGAAAAT	NM_053653.2
R-VEGFC-A			ACAGTCCCGGATCACAATGCT	
R-FOXC2-S	200	60	AAGTGGTGGTCAAGAGCGAG	NM_001101680.1
R-FOXC2-A			CATGATGGTCTCCACGCTGA	
R-GAPDH-S	138	60	CTGGAGAAACCTGCCAAGTATG	NM_017008.4
R-GAPDH-A			GGTGGAAGAATGGGAGTTGCT	

Figures

A

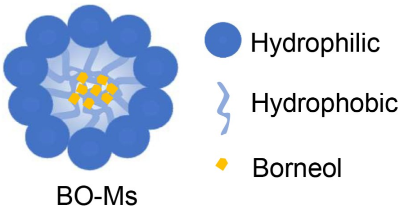

B

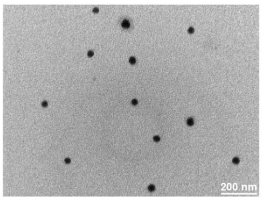

C

	Size (nm)	PDI	Zeta Potential (mV)
BO-Ms	20.06 ± 2.54	0.291 ± 0.012	-2.45 ± 0.87
BL-Ms	17.32 ± 1.49	0.257 ± 0.050	-5.51 ± 0.83

Figure S1. Characterization of BO-Ms and BL-Ms.
(A) Schematic of BO-Ms. **(B)** TEM images of the BO-Ms. (Scale bars, 200 nm). **(C)** Particle diameters and Zeta potential of BO-Ms and BL-Ms. (n = 5, Data are means ± SEM).

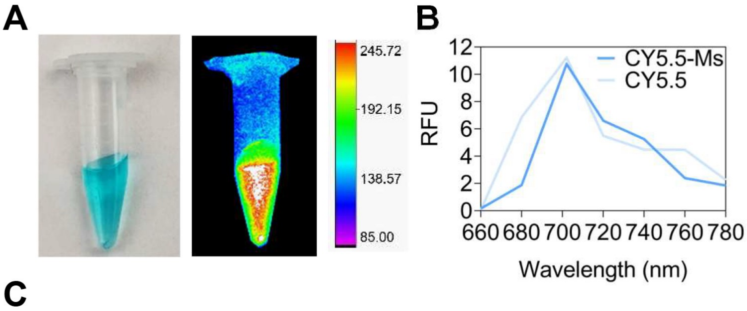

C

	Size (nm)	PDI
CY5.5-Ms	15.12 ± 0.91	0.205 ± 0.041

Figure S2. Characterization of CY5.5-Ms.
(A) Photos of CY5.5-Ms (left) and fluorescence images (right) by an *in vivo* imaging system. (Ex = 610 nm, Em = 700 nm). **(B)** Fluorescence emission spectra of CY5.5-Ms. (Ex = 610 nm). **(C)** Particle diameters and PDI of CY5.5-Ms (n = 5, Data are means ± SEM).

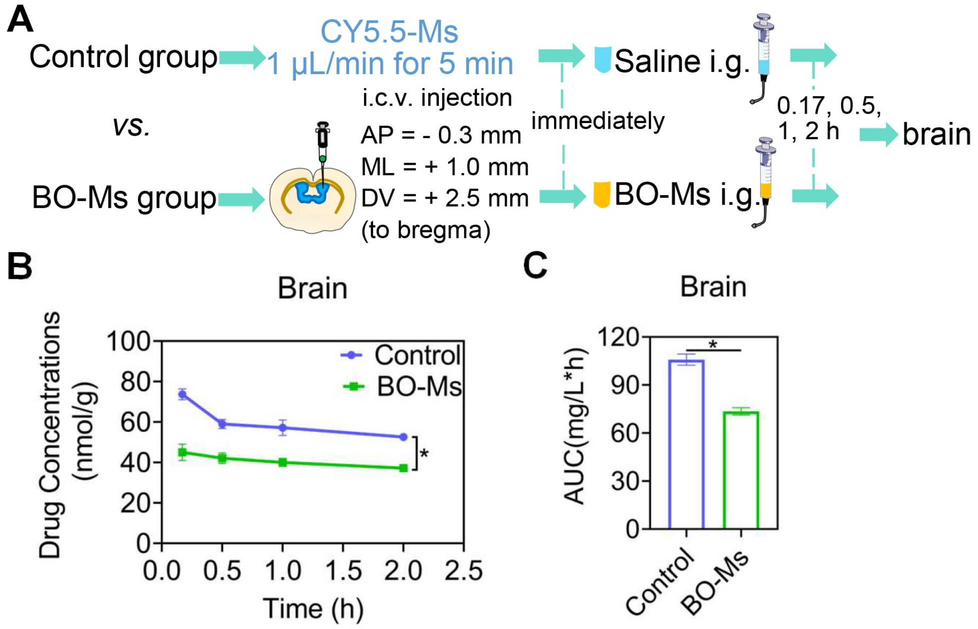

Figure S3. BO-Ms improve the efflux of CY5.5-Ms from the brain.

(A) Schematic of the experimental layout where mice were injected intracerebroventricular (i.c.v.) with CY5.5-Ms and then intragastrical administration (i.g.) of saline or BO-Ms. **(B)** Content of CY5.5-Ms in mice brain in both groups at the 10, 30, 60, and 120 min. *$p < 0.05$ (Student's *t*-test), n = 3-4 mice per group, representative of two independent experiments. Data are means ± SEM. **(C)** $AUC_{(mg/L*h)}$ of CY5.5-Ms in the brain in the control group and BO-Ms group. *$p < 0.05$ (Student's *t*-test), n = 3-4 mice per group, representative of two independent experiments. Data are means ± SEM.

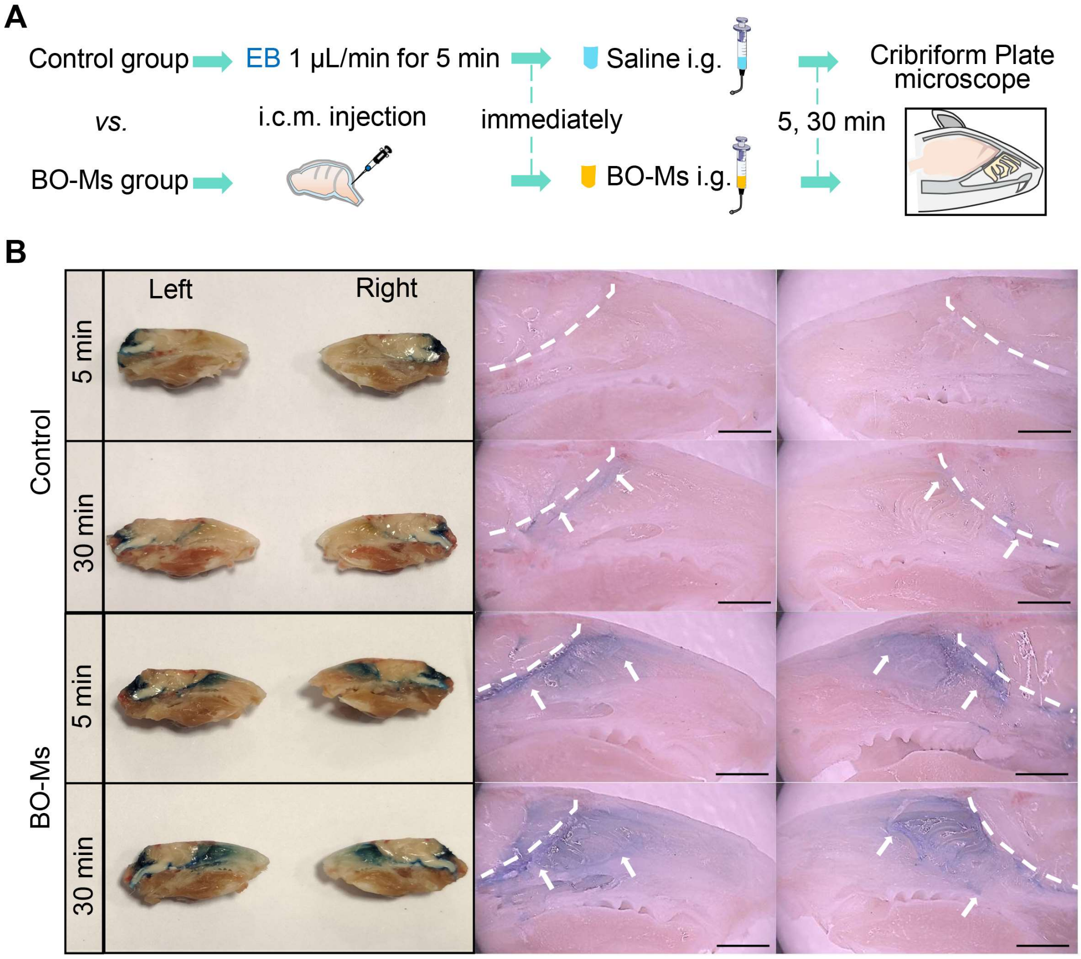

Figure S4. BO-Ms enhance CSF flow into the nasal cavity.

(A) Schematic of the experimental layout where mice were injected the cisterna magna (i.c.m.) with EB and then intragastrical administration (i.g.) of saline or BO-Ms. **(B)** Representative images of the drainage of EB through the cribriform plate into the nasal cavity in the control group and BO-Ms group. (Scale bars, 1 mm).

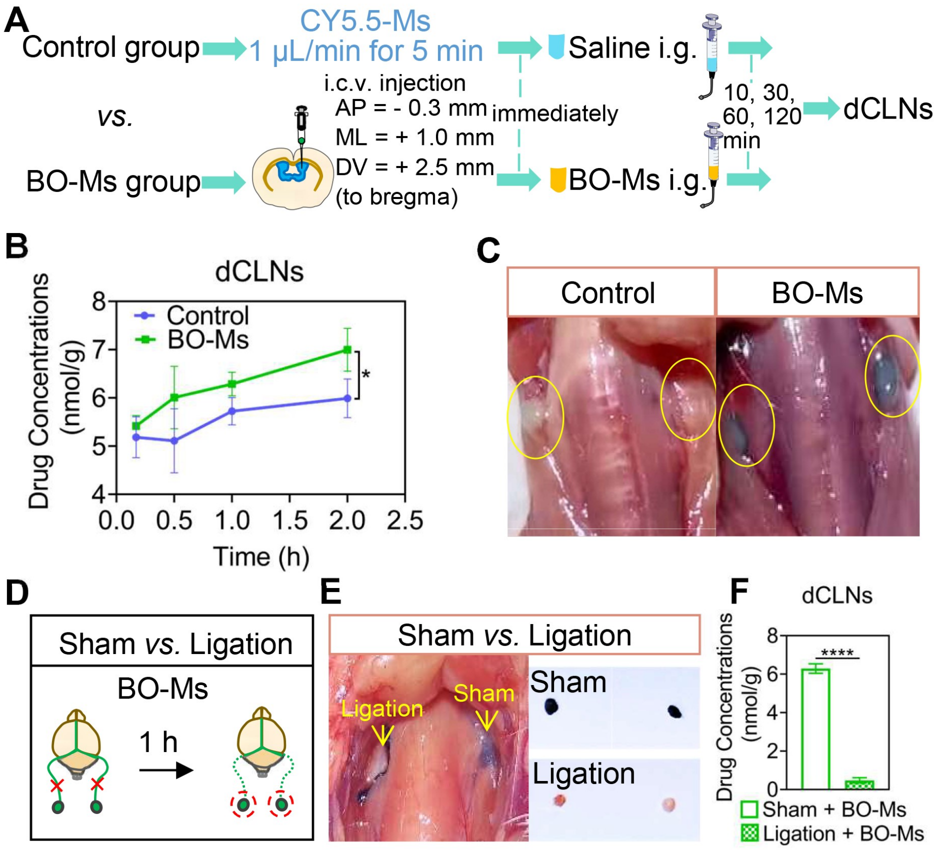

Figure S5. BO-Ms clear the CY5.5-Ms from the CNS into dCLNs via meningeal lymphatics.

(A) Schematic of the experimental layout where mice were injected intracerebroventricular (i.c.v.) with CY5.5-Ms and then intragastrical administration (i.g.) of normal saline as a control group or BO-Ms. The dCLNs were harvested at various times after administration. **(B)** Content of CY5.5-Ms in mice dCLNs in control and BO-Ms groups at the 10, 30, 60, and 120min. *$p < 0.05$ (Student's *t*-test), n = 3-4 mice per group, representative of two independent experiments. Data are means ± SEM. **(C)** Representative images of the dCLNs in the control group and BO-Ms group which 1 h after CY5.5-Ms i.c.v. injection and then the saline or BO-Ms were orally administered. **(D)** Schematic of the experimental ligation of the afferent lymphatic vessel of the dCLNs. **(E)** Representative images of the ligation surgery. Sham-operated or ligated animals were injected with 5 μl of CY5.5-Ms in the lateral ventricle and then oral administration of BO-Ms. The dCLNs were harvested 1 h after BO-Ms oral gavage. Representative images of the CY5.5-Ms accumulation in the dCLNs of the sham-operated group and ligated group. **(F)** Quantity of CY5.5-Ms in the dCLNs in BO-Ms sham group and BO-Ms ligation group 1 h after CY5.5-Ms i.c.v. injection and then the BO-Ms were orally administered. ****$p < 0.001$ (Student's *t*-test), n = 3-4 mice per group, representative of two independent experiments. Data are means ± SEM.

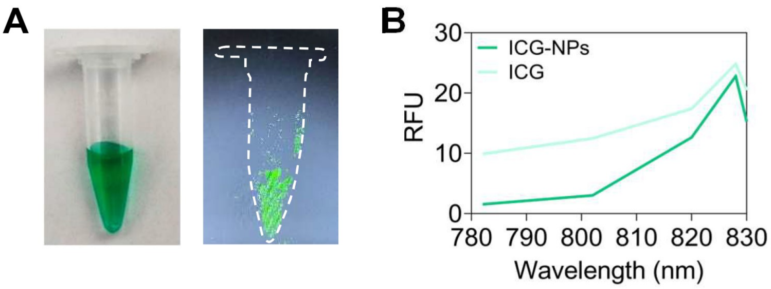

C

	Size (nm)	PDI
ICG-NPs	201.93 ± 4.52	0.262 ± 0.037

Figure S6. Characterization of ICG-NPs.

(A) Photos of ICG-NPs (left) and fluorescence images (right) by a near-infrared fluorescence imaging system of DPM. **(B)** Fluorescence emission spectra of ICG-NPs. (Ex = 760 nm). **(C)** Particle diameters and PDI of ICG-NPs (n = 5, Data are means ± SEM).

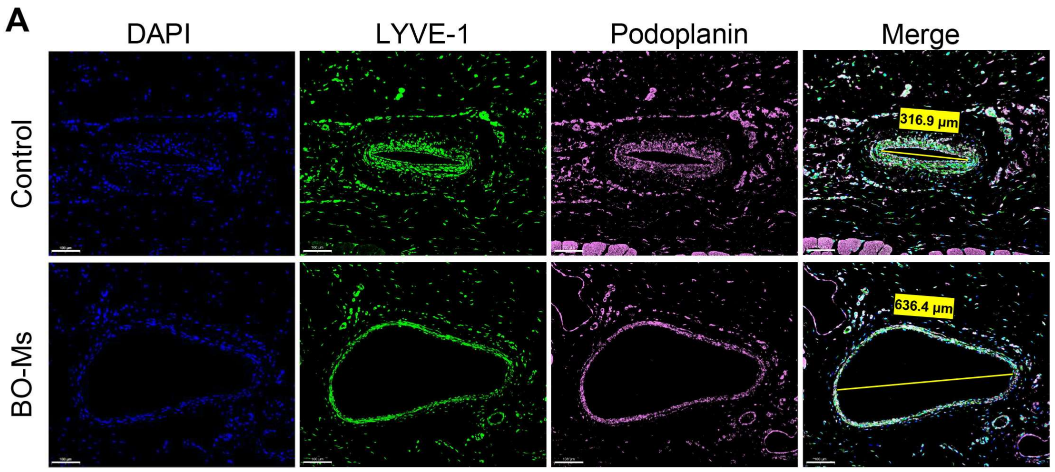

Figure S7. Effect of BO-Ms on the changes in the lymphatic vessels morphology.

(A) Representative image of lymphatic vessels stained with DAPI (blue), anti-LYVE-1 antibody (green), and anti-Podoplanin-antibody (pink) in the control group and BO-Ms group. (Scale bars, 100 μm).

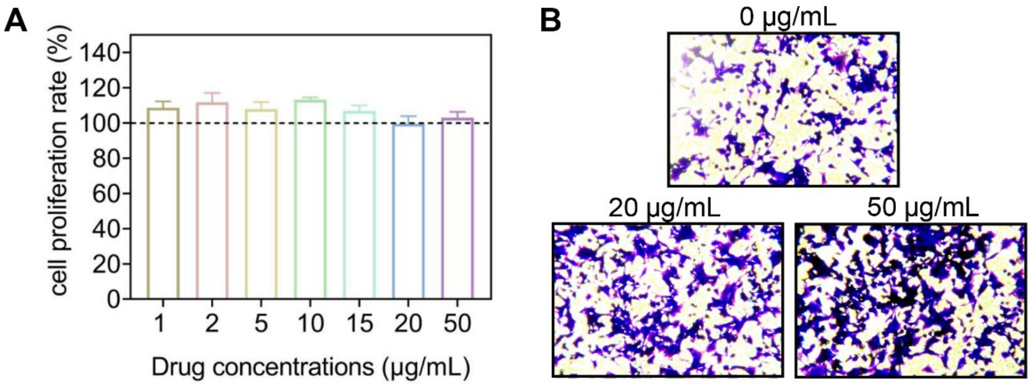

Figure S8. Effects of borneol on proliferation and migration of MLECs. **(A)** Proliferation rate of MLECs with different concentrations of borneol (1, 2, 5, 10, 15, 20, and 50 μg/mL). **(B)** Representative imaging of MLECs migration at different concentrations of borneol (0, 20, 50 μg/mL) stained by crystal violet.

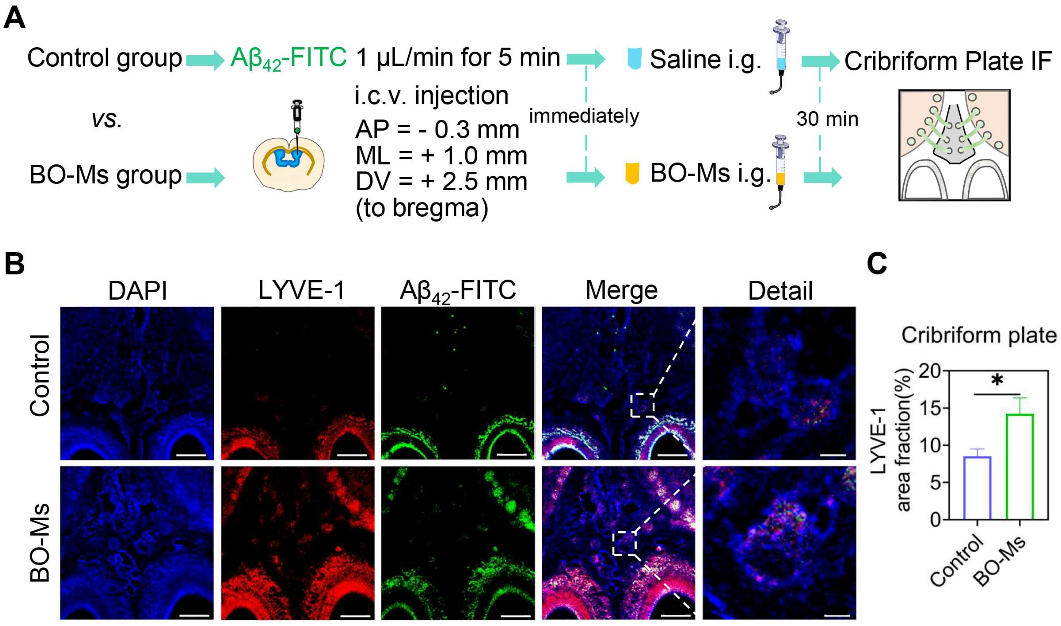

Figure S9. BO-Ms enhance Aβ42 across the cribriform plate.

(A) Schematic of the experimental layout where mice were injected the intracerebroventricular (i.c.v.) with Aβ42-FITC oligomers and then intragastrical administration (i.g.) of saline or BO-Ms. **(B)** Representative cribriform plate stained with DAPI (blue) and anti-LYVE-1 antibody (red) showing Aβ42-FITC oligomers (green) distribution in the cribriform plate vessels of the control group and BO-Ms group at 30 min. (Scale bars, 100 μm. Details figures scale bars, 20 μm). **(C)** Quantification of the percent area coverage of LYVE-1 antibody staining in the cribriform plate. *$p < 0.05$ (Student's *t*-test), n = 3 mice per group. Data are means ± SEM.

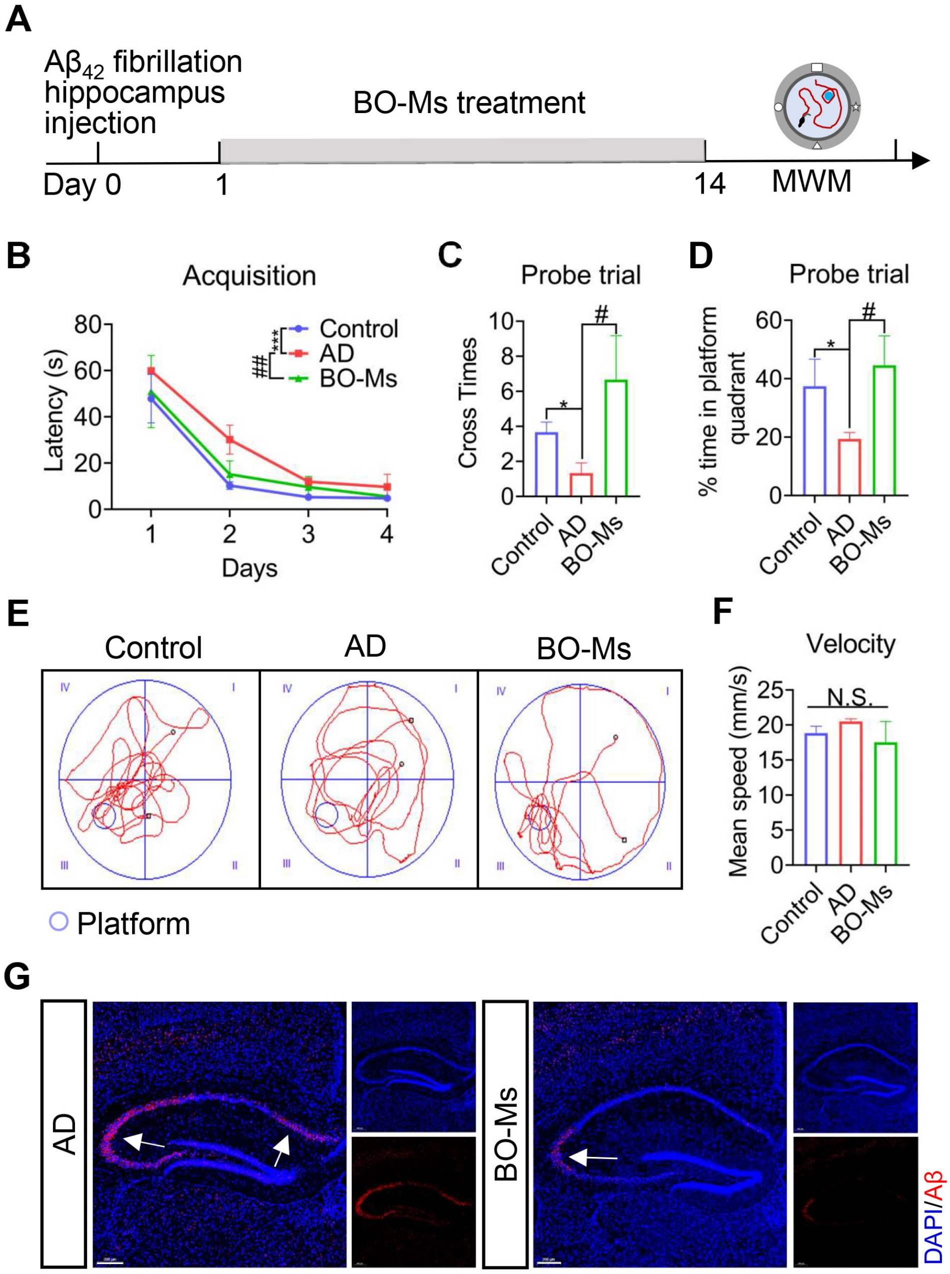

Figure S10. BO-Ms improved memory deficits in AD mice induced by $A\beta_{42}$ fibrillation.

(A) Timeline of BO-Ms treatment in AD mice induced by $A\beta_{42}$ fibrillation. **(B)** Escape latency to the platform during the training trails in an MWM. $^{***}p < 0.001$, *vs.* control group. n = 6 mice per group. $^{\#\#}p < 0.01$, *vs.* AD group. n = 6 mice per group. Data are means ± SEM. **(C)** The number of target platform crossings in the probe test. $^{*}p < 0.05$, *vs.* control group. n = 6 mice per group. $^{\#}p < 0.05$, *vs.* AD group. n = 6 mice per group. Data are means ± SEM. **(D)** Time spent in the target quadrant in the probe test. $^{*}p < 0.05$, *vs.* control group. n = 6 mice per group. $^{\#}p < 0.05$, *vs.* AD group. n = 6 mice per group. Data are means ± SEM. **(E)** Representative track images of mice in the probe test. **(F)** Mean swimming velocity of mice. N.S. = not significant. **(G)** Representative

images of brain sections from each group were analyzed by DAPI (blue) and Anti-Aβ1-42-antibody (red) immunofluorescence. White arrow: $A\beta_{42}$ deposition. (Scale bars, 200 μm).
